# Surveillance and Control of African Swine Fever in the Early Phase of the COVID-19 Pandemic, March-May 2020: A Multi-Country E-Survey

**DOI:** 10.3389/fvets.2022.867631

**Published:** 2022-06-06

**Authors:** Amélie Desvars-Larrive, Annemarie Käsbohrer

**Affiliations:** ^1^Institute of Food Safety, Food Technology and Veterinary Public Health, Unit of Veterinary Public Health and Epidemiology, University of Veterinary Medicine, Vienna, Austria; ^2^VetFarm, University of Veterinary Medicine, Pottenstein, Austria; ^3^Complexity Science Hub, Vienna, Austria

**Keywords:** COVID-19, veterinary services, surveillance, African swine fever (ASF), questionnaire, preparedness, animal health

## Abstract

Stringent COVID-19 public health and social measures (PHSMs) have challenged the work of animal health professionals, especially in the early phase of the pandemic. We aimed to qualitatively describe how COVID-19 PHSMs have affected the surveillance and control of African swine fever (ASF) in Europe, assess how professionals engaged in these activities perceived the impact of the COVID-19 crisis, and identify potential areas of improvement. An online questionnaire was proposed *via* email between 9 December 2020 and 22 January 2021 to professionals engaged in ASF-related activities in Europe and Eastern neighboring countries. The questionnaire contained questions pertaining to ASF surveillance and control activities between March and May 2020, respondent's perception of the impact of COVID-19 PHSMs on these activities, and respondent's opinion on potential improvements to prepare for future crises. Economic and sanitary variables were used to describe the national contexts over the study period. Twenty-seven respondents from 24 countries participated to the study. Essential activities related to surveillance and management of ASF were reduced and/or adapted but maintained in most surveyed countries. Communication was mentioned as the first area of improvement during crisis while maintenance of efficient veterinary services and surveillance activities were cited second and third top priorities. The need for the development of remote procedures was also recognized. Some respondents highlighted difficulties in ensuring biosecurity and biosafety of the field actors due to shortage in protective equipment. Only a small majority (52%) of the survey participants agreed that their institution/working group is better prepared to future lockdown-type situations. Our study emphasizes that short-term measures were globally successful to tackle the immediate impacts of the COVID-19 crisis on the routine duties of professionals involved in ASF surveillance and control. Our findings suggest that country-specific improvements are necessary to support and advance the preparedness of the actors involved in infectious animal disease surveillance and control in case lockdown-like measures are implemented. Overall, our results highlight the crucial importance of recognizing animal health services as essential activities during crisis.

## Introduction

Since January 2020, in response to the COVID-19 pandemic (1), governments worldwide have implemented a wide range of public health and social measures (PHSMs) aiming at mitigating the spread of the virus (2, 3). Over the period March-May 2020, most European countries had declared the state of emergency and were experiencing their first national lockdown, i.e., implementing large scale physical distancing measures and movement restrictions (4), including most of the time a stay-at-home order (confinement). The global situation was unprecedented and the epidemiological context highly unstable. Human activities/businesses were reduced to those considered as essential. These PHSMs, although crucial to curb the spread of COVID-19, had negative impacts on the society, the economy, the environment (5–7), and global human health [e.g., mental health burden (8), postponement of non-urgent cares, disruption of cancer treatments (9, 10) and health program activities (11), hesitation of patients to consult due to fear of COVID-19 (12, 13)].

Animal production and health services were heavily affected by the global crisis. COVID-19 clusters have been found in slaughterhouses and meat processing plants in various countries (14–17), inducing a negative impact on the livestock supply chain (18, 19). Closure of restaurants and prohibition of organized events have led to a decrease in demand and change in consumption patterns of products of animal origin (20, 21). Moreover, COVID-19 put unprecedented stress on the supply of masks and other personal protective equipment (PPE) (22, 23), hand sanitizer, cleaning products, and disinfectants, which were mainly redirected toward human healthcare settings, leading to a shortage of critical PPE and disinfection supplies in the animal health sector (23). Global shortages of laboratory consumables and reagents (24) have also affected veterinary diagnostic capacity while several veterinary laboratories worldwide were repurposed to process human samples for COVID-19 testing (25–27). Under these circumstances, the World Organisation for Animal Health (OIE) and the World Veterinary Association (WVA) jointly declared veterinary services to be “essential activities” (28) while a communication of the European Commission on the implementation of “green lanes” stated that veterinary medicines were essential goods (29).

African swine fever (ASF) is a transboundary animal disease (TAD) affecting species of the Suidae family. This viral disease is notifiable in the European Union (EU) and to the OIE and its control is governed by EU and national legislations (30, 31). The genotype I ASF virus (ASFV) was first reported in Portugal in 1957 before spreading to Western European countries: Spain (1960), France (1964), Italy (1967), Malta (1978), Belgium (1985), and the Netherlands (1986) (32, 33). European countries successfully eradicated the disease in 1995, with the exception of the island of Sardinia, Italy, where the genotype I ASFV has been endemic since 1978 (34, 35). Genotype II ASFV was introduced in Europe, in Georgia, in 2007 (36) and then slowly spread to neighboring countries (Armenia, Azerbaijan, Russia, and Belarus). In 2014, the first cases in wild boars were reported in Lithuania. Cases were subsequently reported in Estonia, Latvia, and Poland, more recently in the Czech Republic (2017), Romania (2017), Hungary (2018), Bulgaria (2018), Belgium (2018), and Slovakia (2019) (32). In 2020, Germany reported its first case in a wild boar (37). This year (2022), the disease was reported in the north of Italy. In the absence of treatment or vaccine, controlling the spread of the disease can only be achieved by the strict implementation and supervision of recommended measures. Preventive measures include, among others, the control of wild boar density through hunting, the active search for wild boar carcasses, strict ban on swill feeding, collection of rubbish material on roads, in the forests and parks to reduce the exposure of wild boars, increase of biosecurity measures in pig farms, and border controls. Passive surveillance in domestic pigs and wild boars (i.e., investigation of wild boars found sick or dead) is one of the most important tool for early detection of ASF (38).

On the frontline of ASF, during the first national “lockdowns” (or when “lockdown-like” measures where enforced) in March-May 2020, prohibition of recreational hunting, shortage of qualified personnel, protective equipment and laboratory supplies, or remote work could have particularly challenged the “routine” work of veterinary services and other stakeholders involved in pig production and wild boar management. A rapid assessment of the challenges faced by the professionals involved in the surveillance and management of infectious animals is critical at the early stage of a crisis for a rapid adjustment of the workforce and material supply to the real needs in the field. Using an online questionnaire, our study aimed to i) describe qualitatively how COVID-19 PHSMs have affected the surveillance and control of ASF in Europe and Eastern neighboring countries during the early phase of the pandemic; ii) describe adaptive measures implemented in the different countries to ensure ASF-related activities; iii) assess how professionals engaged in the surveillance and control of ASF perceived the impact of the COVID-19 crisis on the ASF epidemic in their country; and iv) identify potential areas of improvement that could be considered by veterinary authorities preparing for future crises.

## Materials and Methods

### Questionnaire

The questionnaire (Supplementary Material 1) was developed in English and distributed using Google Forms. The questionnaire was reviewed and pre-tested by two researchers and one student. Submitting a response did not require a log-in. Invitations to participate in the study (including the link to the Google Form) was sent by email on 9 December 2020 to 179 Chief Veterinary Officers (CVOs) and animal health professionals whose work was related to ASF in Europe (44 countries) and Eastern neighboring countries (six countries, see https://euneighbours.eu/en). Email addresses were retrieved from public webpages and documents. Our objective was to obtain, at least, one answer per country. Participants were able to access the survey and complete it on a computer or a mobile device. The questionnaire in PDF format was emailed to the survey participants on demand. A reminder was sent on 10 January 2021 and responses were collected until 22 January 2021.

The survey entry page described the background and objectives of the study and provided the coordinates of the contact person. The second page displayed information related to privacy policy (39). The questionnaire itself consisted in six different sections:

1. The first section (seven items) aimed at characterizing the respondent;
2. The second section (15 items) aimed at assessing how COVID-19 PHSMs may have affected hunting activities and wild boar management;
3. The third section (two items) aimed at assessing how COVID-19 PHSMs may have affected the activities of official veterinarians and swine veterinary practitioners;
4. The fourth section (14 items) aimed at assessing how COVID-19 PHSMs may have affected ASF management and regulations, including impact on ASF training procedures, border controls, and laboratory activities;
5. The fifth section provided insights into how respondents perceived the impact of the COVID-19 crisis on the surveillance and control of ASF in their countries (five items). A five-level Likert scale (1 = strongly disagree, 2 = disagree, 3 = neutral, 4 = agree, or 5 = strongly agree) was used to rate the degree to which respondents agree or disagree with each statement. Two supplementary items targeting respondents whose country reported ASF cases before March 2020 aimed to measure how they perceived the impact of the COVID-19 crisis on the further spread of ASF in their countries;
6. The sixth section (one item) aimed at surveying the respondents' opinion on potential areas of improvement that the veterinary authorities could envisage to be better prepared for future crises.

In the questionnaire, we used the term “feral pig,” i.e., a pig that is not kept or bred on a holding as defined in the Council Directive 2002/60/EC (30) (therefore including “wild boar”). However, because the term “wild boar” is more commonly used in the context of ASF, this term has been preferred throughout this paper.

### Indicators of the Sanitary Situation and Economic Context

To provide a broad view of the study period March-May 2020, we used a set of seven indicators to capture the multiple dimensions of the economic context and sanitary situation (i.e., related to COVID-19 and ASF) in the responding countries: i) The average wild boar density for each country was computed from the gridded data of the predicted wild boar density based on the mosaicked model from Pittiglio et al. (40); ii) The average domestic pig density of the country was computed using the data extracted from the Gridded Livestock of the World (GLW 3) database (https://dataverse.harvard.edu/dataverse/glw_3, accessed on 15 March 2021) for the year 2010 (41); iii) The numbers of new ASF cases in wild boars and iv) in domestic pigs, per country, over the study period (March-May 2020), were extracted from the FAO's EMPRES-i website (http://empres-i.fao.org/, accessed on 10 June 2020); v) The COVID-19 cumulated number of confirmed deaths per million inhabitants per country over the study period March-May 2020 and vi) the gross domestic product (GDP) per capita for each country were retrieved from the GitHub repository of our World in Data (42); vii) The median stringency of the COVID-19 PHSMs over the period March-May 2020 was estimated using the original stringency index provided by the Oxford COVID-19 Government Response Tracker (OxCGRT) (3), which records the strictness of “lockdown style” policies that primarily restrict people's behavior.

### Analytical Strategy

All computations and visualizations were performed in R Studio (43), using R 4.0.3 (44). Standard descriptive statistics were used to describe response frequency. We applied principal component analysis (PCA) (45) to explore associations among economic and sanitary variables of the responding countries during the study period, March-May 2020. The Kaiser criterion was used to determine the number of dimensions to retain for further analysis (i.e., dimensions with eigenvalues ≥1). A hierarchical clustering on principal components (HCPC) using Ward's method (46) was performed subsequently to the PCA to obtain an unsupervised clustering of the responding countries with similar characteristics.

We considered the Likert scale data (fifth section) as ordinal scale data (47, 48). To delineate groups of respondents with a similar perception, we applied multiple correspondence analysis (MCA) to the Likert scale data (46). To decrease the number of categories in the MCA and characterize trends in the respondents' perception, categories “strongly disagree” and “disagree” were merged into the category “disagree” while categories “strongly agree” and “agree” were merged into the category “agree.” A HCPC using Ward's method (46) was then performed to cluster respondents with a similar perception.

The computations of the PCA/MCA was performed using the R packages *FactoMineR* (49) and *factoextra* (50). Missing data for the PCA (stringency index for Armenia, average wild boar density for Israel) was managed using the R package *missMDA* (51).

Responses to the open-ended question in section six (“*In your opinion, what are the three main points the veterinary authorities should focus on in the event of another COVID-19 lockdown/crisis*”) were analyzed using thematic analysis (52), where concepts and patterns of meaning in the data are identified. The three points mentioned by the respondents were considered non-ordered (i.e., point 1 was not considered more priority than point 3). Text responses were manually coded into themes [i.e., patterns of meaning which capture important points in the data (52)] and subthemes using distinct words or short phrases, which revealed different concepts, key words, and ideas.

We used the packages *ggplot2* (53) and *likert* (54) to visualize our data.

### Ethics

The first window of the e-survey consisted of i) an information statement giving potential participants the necessary understanding for the motivation and procedures of the study as well as contact data to answer any further questions and ii) a statement that by filling out and returning the survey, the participants give their informed consent (Supplementary Material 1). As the questionnaire required the collection of data relating to human subjects (e.g., position in their institute), it was submitted to the Ethics Committee of the Medical University of Vienna, Austria. This Ethics Committee decided that an official decision on the present study was not required, in accordance with the current Austrian legislation.

## Results

### Characteristics of the Respondents

**Figure 1:**
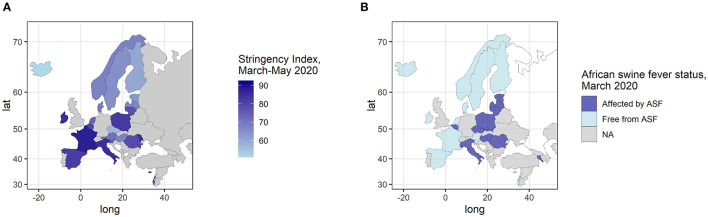
Context of the study in the 24 responding countries. **(A)** Stringency index of the COVID-19 government response over the period March-May 2020 computed from the Oxford COVID-19 Government Response Tracker (OxCGRT) dataset (3) (no data available for Armenia); **(B)** African swine fever status of the 24 responding countries in March 2020 (start of the study period).

Twenty-five percent of the emails (*n* = 44) could not be delivered. Twenty-seven respondents (response rate = 27/135, 20%) from the following 24 countries answered the e-survey (25 *via* the Google Form, two requested a PDF version of it): Armenia, Austria (*n* = 2), Belgium (*n* = 3), Cyprus, Czech Republic, Denmark, Estonia, Finland, France, Hungary, Iceland, Ireland, Israel, Italy, Latvia, Lithuania, Moldova, Netherlands, Norway, Poland, Romania, Slovenia, Spain, and Sweden (Figure 1). We therefore reached 46% (23/50) of the targeted countries and additionally received an answer from Israel, which we included in the study. Twenty-three respondents were veterinary officers (among them two were member of the EU ASF Task Force), two were head of or working in a veterinary laboratory, one was a member of the federal veterinary office, and one was a wildlife expert involved in wild boar-ASF management.

### Sanitary Situation and Economic Context

Stringency of the government policies (3) in the responding countries is presented in Figure 1A. Eleven (45.8%) out of the 24 answering countries had reported ASF cases in domestic pigs and/or wild boars before the study period (March-May 2020) (Figure 1B). Other indicators describing the context in the responding countries over the period March-May 2020, i.e., number of confirmed deaths per million, GDP per capita, density of ASF-susceptible species in the responding countries, are presented in Supplementary Material 2.

During the study period (March-May 2020), five responding countries reported ASF cases in wild boars (Belgium, *n* = 3; Hungary, *n* = 2,987; Latvia, *n* = 68; Poland, *n* = 1,423; and Romania, *n* = 449) while two countries reported ASF cases in domestic pigs (Poland, *n* = 394 and Romania, *n* = 7,366).

**Figure 2:**
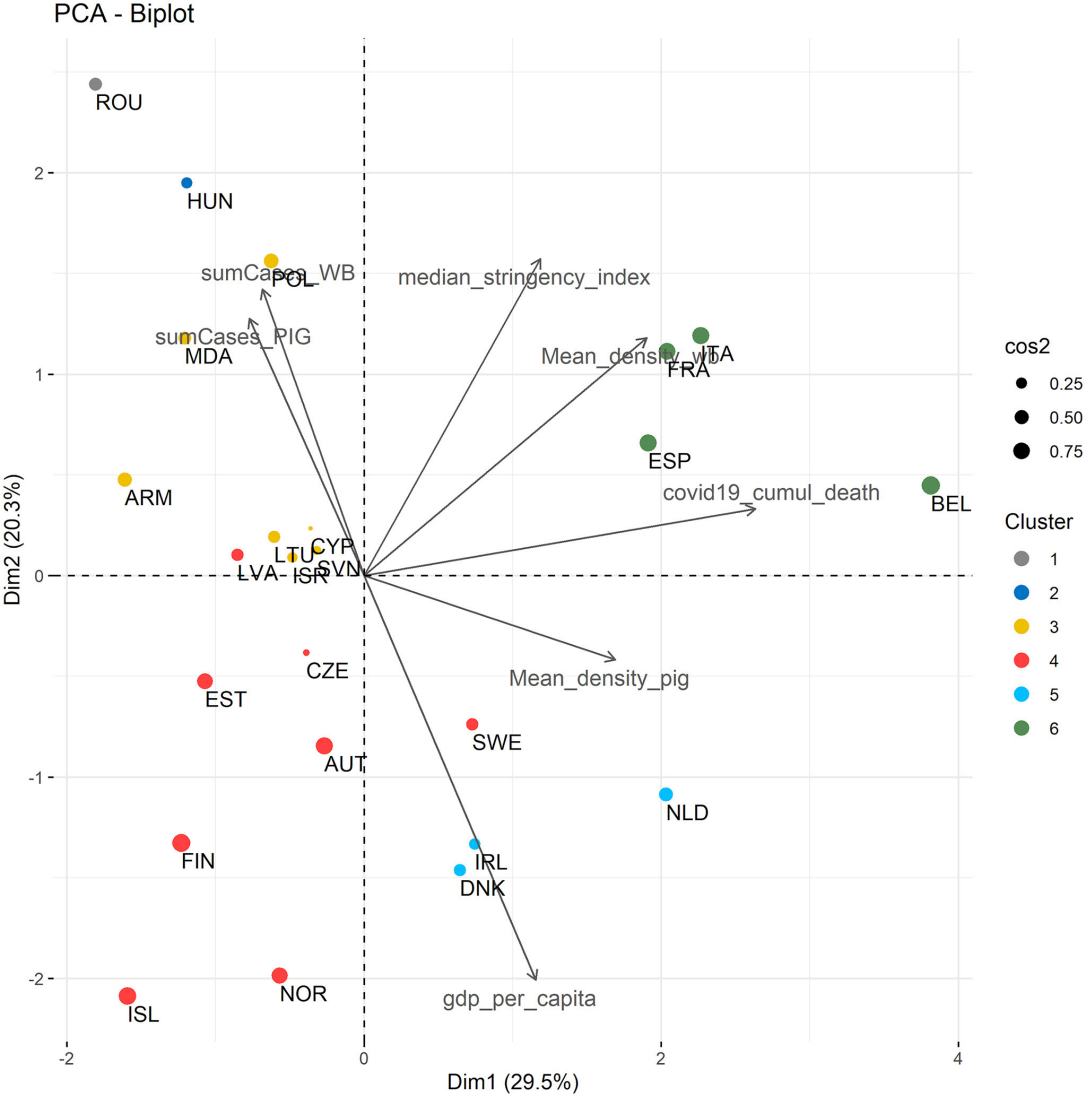
Biplot of the first two axes of the principal component analysis (PCA) showing the clustering of the 24 participating countries based on sanitary and economic variables. Countries are colored according to their cluster. Dot size corresponds to cos2 value. ARM, Armenia; AUT, Austria; BEL, Belgium; CYP, Cyprus; CZE, Czech Republic; DNK, Denmark; ESP, Spain; EST, Estonia; FIN, Finland; FRA, France; ISL, Iceland; ISR, Israel; ITA, Italy; HUN, Hungary; IRL, Ireland; LVA, Latvia; LTU, Lithuania; MDA, Moldova; NLD, Netherlands; NOR, Norway; POL, Poland; ROU, Romania; SVN, Slovenia; SWE, Sweden.

The PCA identified three significant dimensions (Dim) accounting for a total of 64.8% of the variance in the dataset. The most weighted contributions to Dim1 were the cumulated number of COVID-19 confirmed deaths per million (March-May 2020) and the mean density of wild boars. The most weighted contributions to Dim2 were the GDP per capita and the median stringency index (March-May 2020) (Supplementary Material 3). The HCPC identified six clusters of countries (Figure 2) with similar sanitary and economic characteristics over the period March-May 2020. As indicated by the metrics in Supplementary Material 4, Cluster 1 (Romania) is defined by a number of ASF cases in domestic pigs that is greater than average over the study period, compared to the other responding countries. Cluster 2 (Hungary) is defined by a higher number of reported ASF cases in wild boars over the study period. Cluster 3 (Armenia, Cyprus, Israel, Lithuania, Moldova, Poland, and Slovenia) is mostly characterized by a relatively high median stringency index (of COVID-19 implemented measures) and a lower GDP per capita. Cluster 4 (Austria, Czech Republic, Estonia, Finland, Iceland, Latvia, Norway, and Sweden) is defined by a lower median stringency index than average among the responding countries. Cluster 5 (Denmark, Ireland, and the Netherlands) is characterized by a higher mean density of domestic pigs and a high GDP per capita. Cluster 6 (Belgium, France, Italy, and Spain) is defined by a higher number of cumulated COVID-19 confirmed deaths over the study period, a relatively high average wild boar density, and a relatively high median stringency index over the study period.

### Responses to the Questionnaire

Descriptive summaries of the answers to sections two to four of the questionnaire are displayed in Supplementary Materials 5, 6.

#### Hunting Activities and Wild Boar Management

**Figure 3:**
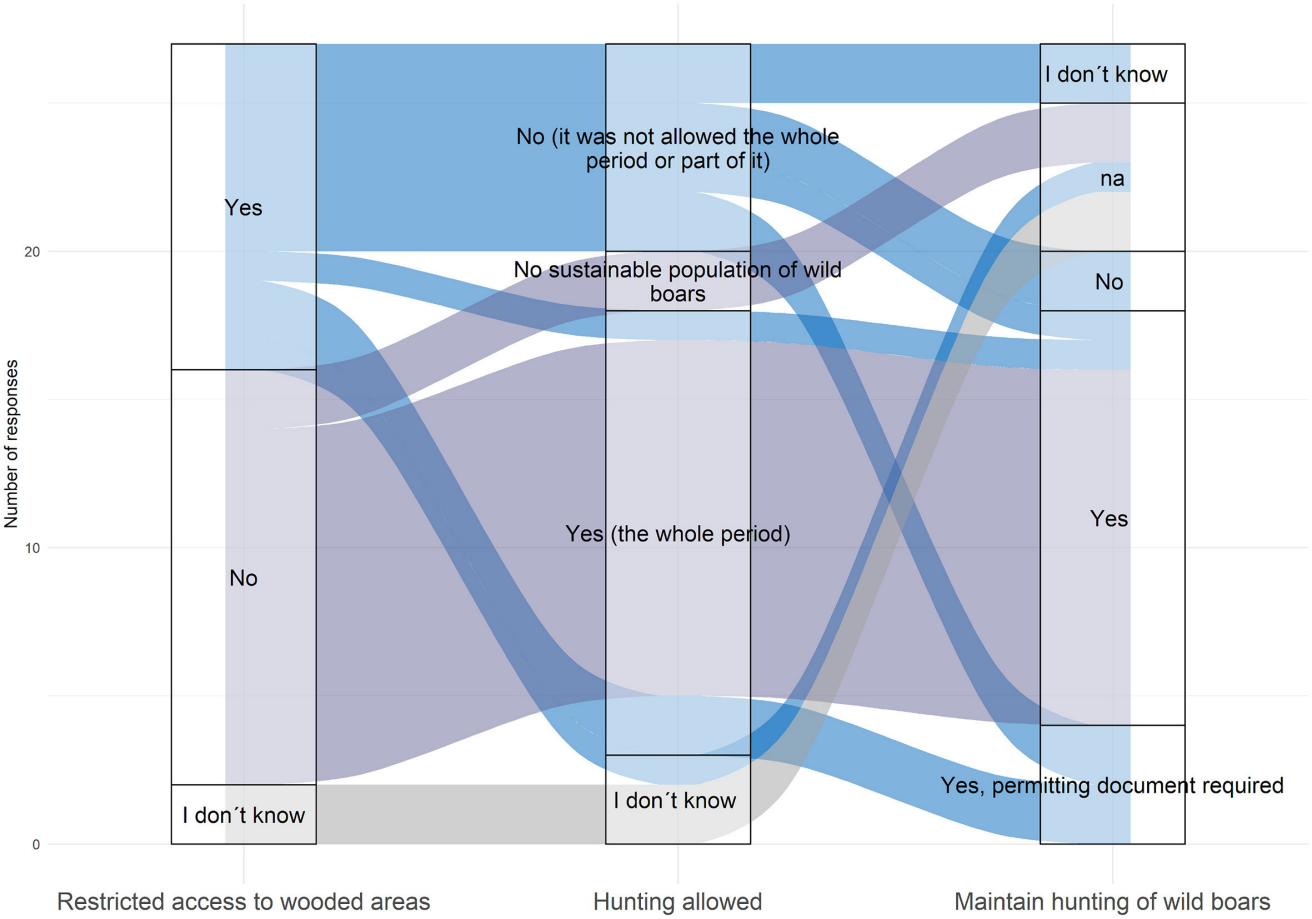
Sankey diagram showing different modalities and adaptation of the wild boar hunting activities, over the period March-May 2020, as reported by the 27 survey participants from 24 countries (na: no answer).

Eleven respondents (40.7%), belonging to 11 different countries, reported access restrictions to wooded areas or national parks. Fourteen respondents (51.8%) reported maintenance (sometimes partial) of hunting activities (any game) in March-May 2020 (Figure 3). Over the year 2020, hunting was restricted from 11 to 305 days (five answers). In four countries, permitting documents exempting some hunters from the stay-at-home order were delivered.

Three respondents out of the four (75%) who declared that their country usually manages winter feeding areas for wild boars, answered that those feeding areas could be maintained over the period March-May 2020. Eight respondents (29.6%), from eight countries, reported that active search for wild boar carcasses is a usual surveillance tool in their country. Among them, six (75%) could maintain this activity in March-May 2020. In two countries, adaptive procedures were implemented, i.e., reduction in the number of persons involved in carcass searches, delivery of permitting documents for official veterinarians and forestry workers, and use of personal protective equipment.

#### Activities of Official Veterinarians and Swine Veterinary Practitioners

**Figure 4:**
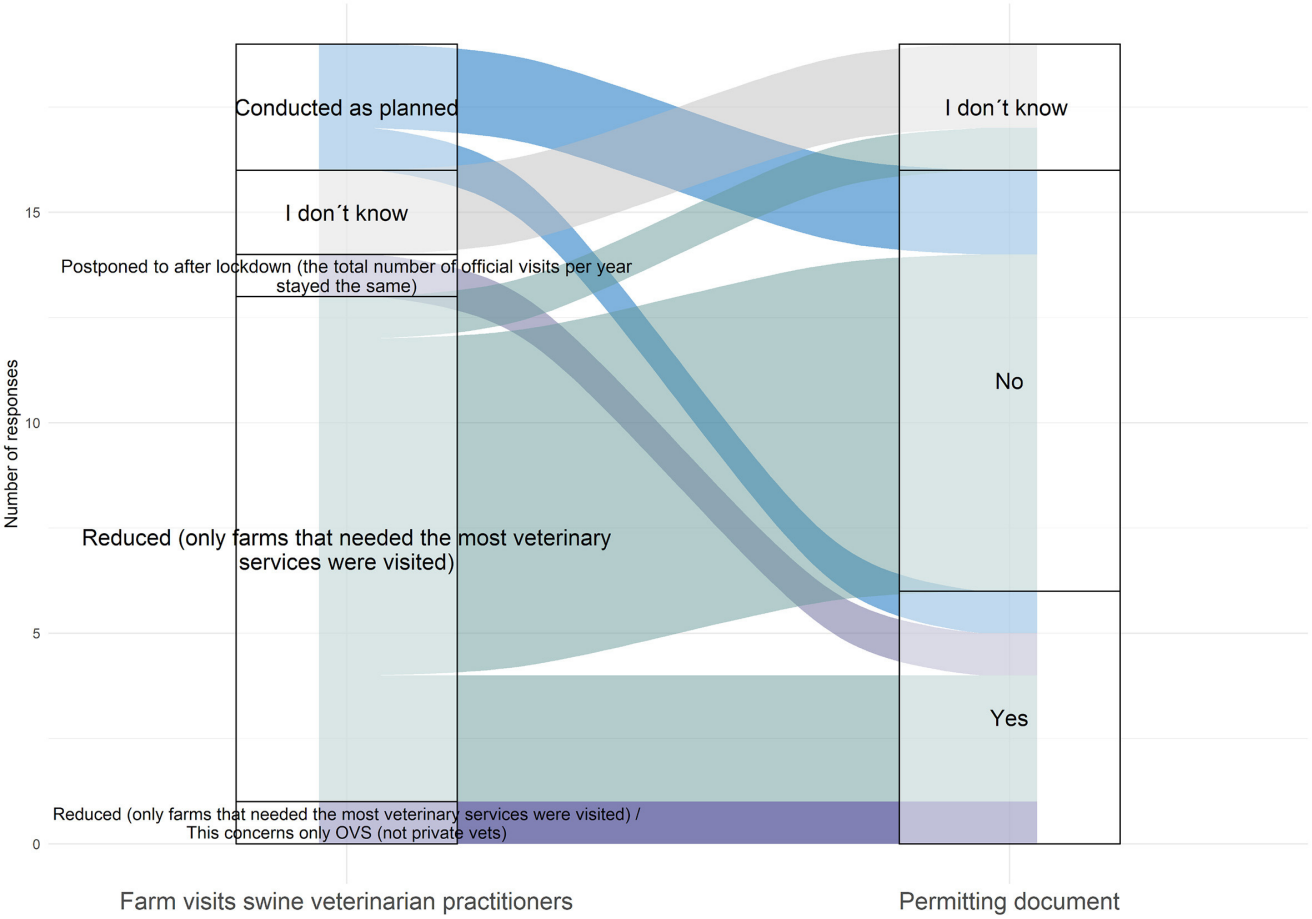
Sankey diagram showing different modalities and adaptations of the swine veterinary practitioner activities, over the period March-May 2020, as reported by 19 survey participants from 18 countries (OVS: Official VeterinarianS).

Eight respondents (33.3%) from six countries skipped this section. Thirteen respondents (68.4%) reported reduction in veterinary visits of pig farms in their countries, i.e., only essential veterinary services were provided or visits were postponed until after lockdown (one country). Three (15.8%) respondents declared no disruption in the routine veterinary farm visits. Movement permits were necessary for veterinarians in six (31.6%) countries (Figure 4).

#### African Swine Fever Training Procedures, Border Controls, and Laboratory Activities

**Figure 5:**
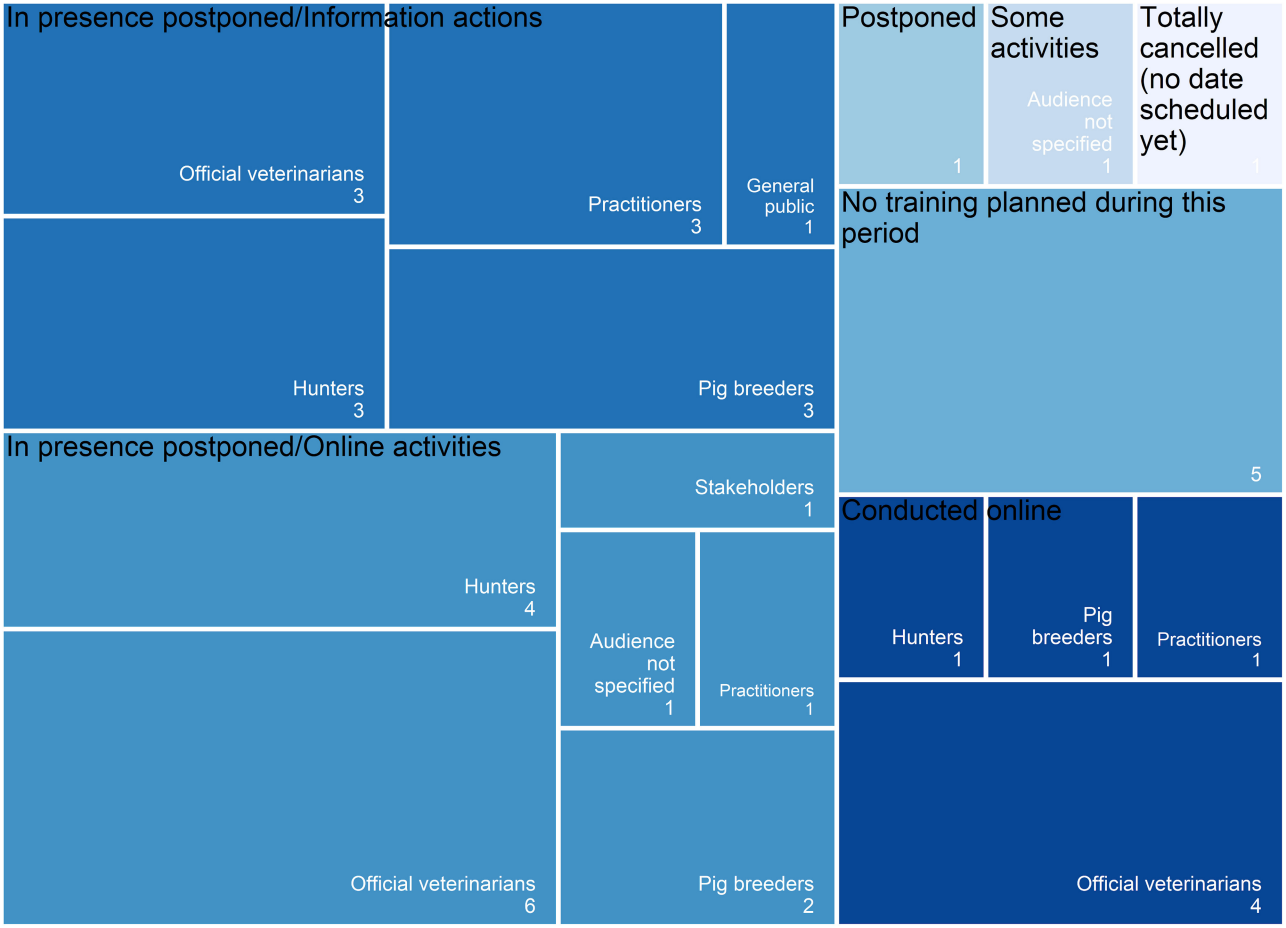
Tree map visualization of the training activities on African swine fever and targeted audience of the online activities over the period March-May 2020, as reported by the 27 respondents from 24 countries. Size of the squares is proportional to the number of answers (indicated in the bottom right corner).

Between March and May 2020, to the respondents' knowledge, the training procedures related to ASF for, e.g., veterinarians, breeders and hunters, were maintained and conducted online in four (17%) countries, mostly for official veterinarians. In 11 (40.7% of the respondents) countries, in-presence training was postponed but online information actions were carried out, e.g., for official veterinarians, practitioners, pig breeders, hunters, the public (including travelers), or stakeholders. African-swine fever-related training was postponed (without any online actions in the meantime) or totally canceled (i.e., not rescheduled at time of survey) in one country each. Three countries (corresponding to four respondents, 14.8%) adapted in-presence training procedures to the COVID-19 measures (e.g., physical distancing, reduced number of persons). Five (21%) countries had no training planned during the study period (Figure 5).

One respondent indicated having experienced delays in ASF laboratory diagnostic tests due to staffing shortage and because the veterinary laboratory was repurposed to process human samples for COVID-19 testing. No respondents mentioned closure of rendering plants in March-May 2020. Twenty-three (85.2%) respondents reported that border control measures for ASF were implemented as usual between March and May 2020 whereas three (11.1%) mentioned that the controls were less regular (two respondents from the same country provided different answers: “as usual” vs. “less regular”).

Among the 13 survey participants belonging to countries affected by ASF as of March 2020 (*n* = 11), one (7.7%) declared that COVID-19 PHSMs have affected the response time of the veterinary authorities when ASF was suspected in a pig farm (i.e., the time elapsed between suspicion by a practitioner and visit by an official veterinarian was increased) and have also delayed the subsequent implementation of the contingency plan. Two (18.2%) respondents reported a delay in the implementation of the contingency plan after a diagnosis of ASF in a wild boar. In all cases, the impact of the delay was considered as minor.

#### Perception of the Impact of the COVID-19 Crisis on the Surveillance, Control and Potential Spread of ASF

**Figure 6:**
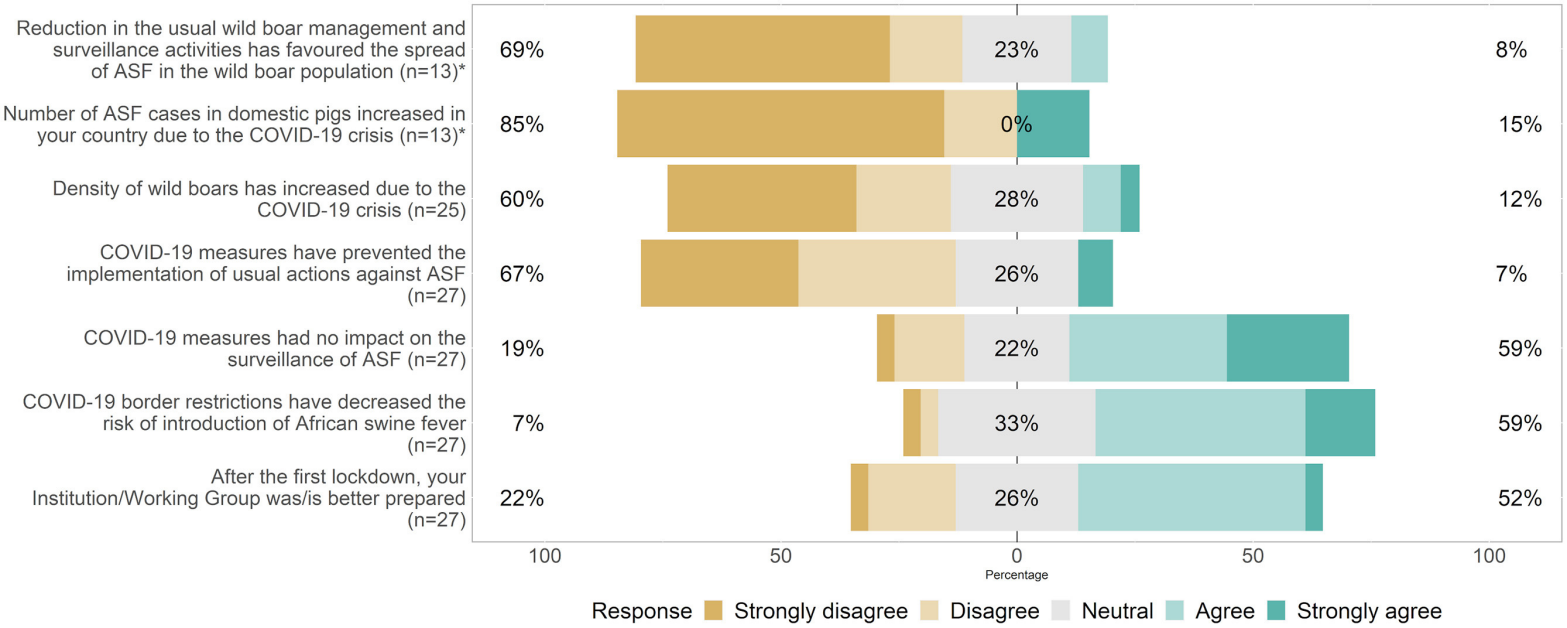
Respondents' (*n* = 27) perception of the impact of the COVID-19 crisis on ASF surveillance and control activities and further spread in their countries (the two items at the top of the figure, marked with an asterisk, targeted respondents whose country reported ASF cases before March 2020).

Most respondents (60%) believed that the COVID-19 PHSMs enforced in March-May 2020 did not lead to an increase in the wild boar density in their country. Two third of the respondents declared that the COVID-19 restrictions did not hamper the implementation of the usual actions against ASF (7% believed the opposite). Similarly, 59% of them considered that the COVID-19 policies had no impact on the ASF surveillance activities (19% perceived an impact on it). Sixteen respondents (59%) agreed that COVID-19 border restrictions implemented in March-May 2020 have decreased the risk of introduction of ASF in their country (7% disagreed). A small majority of the respondents (52%) thinks that their institution/working group is better prepared to work under lockdown-type situations in the future. The majority (69%) of the respondents from countries affected by ASF prior to the COVID-19 crisis (*n* = 13) did not identify reduction in the usual wild boar management and surveillance activities as a facilitator of ASF spreading in the wild boar population. Most of them (84.6%) did not perceive the COVID-19 crisis as a cause of increased ASF cases in domestic pigs (Figure 6).

**Figure 7:**
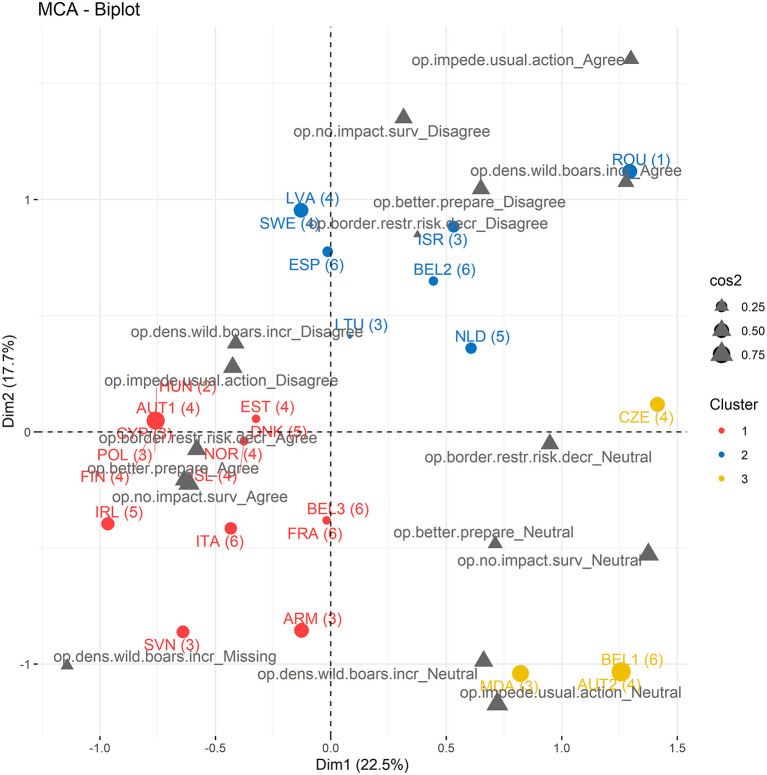
Biplot of the first two axes of the multiple correspondence analysis (MCA) showing the clustering of the 27 survey participants based on their answers to the section five of the questionnaire (Likert-scale response categories). Survey participants are colored according to their cluster. Numbers in brackets correspond to the cluster numbers grouping countries based on sanitary and economic variables (PCA-HCPC, Figure 2). Dot size corresponds to cos2 value. ARM, Armenia; AUT, Austria; BEL, Belgium; CYP, Cyprus; CZE, Czech Republic; DNK, Denmark; ESP, Spain; EST, Estonia; FIN, Finland; FRA, France; ISL, Iceland; ISR, Israel; ITA, Italy HUN, Hungary; IRL, Ireland; LVA, Latvia; LTU, Lithuania; MDA, Moldova; NLD, Netherlands; NOR, Norway; POL, Poland; ROU, Romania; SVN, Slovenia; SWE, Sweden (number attached to some country ISO corresponds to respondent number when more than one respondent from a country answered to the questionnaire).

The first four dimensions of the MCA explained 64.6% of the variance in the Likert scale data (Supplementary Material 7). The variables that contributed most to the first dimension of the MCA (accounting for 22.5% of the variance) were the respondents' perception on the impact of COVID-19 PHSMs on the ASF surveillance activities (eta2 = 0.66) and on the risk of ASF introduction in their countries (eta2 = 0.51). In the second dimension (17.7% of the variance), they were the respondents' perception on the impact of COVID-19 PHSMs on the usual actions implemented against ASF (eta2 = 0.60) and on the density of wild boars (eta2 = 0.54) (Supplementary Material 8). The cluster analysis on the first four dimensions of the MCA object identified three clusters of respondents (Figure 7). Cluster 1 (center left on the biplot) groups survey participants who believed that the COVID-19 PHSMs did not affect the activities of ASF surveillance in their country, that COVID-19 border restrictions decreased the risk of ASF introduction in their countries, and that their institutions/working groups are better prepared to face a similar crisis. Most respondents which country of origin shows a high GDP per capita, lower stringency index in March-May 2020, lower average wild boar density, and absence of ASF cases as of March 2020 (cluster 4, Figure 2) tend to be grouped in cluster 1 by the MCA-HCPC (exception: respondents from Hungary, Belgium #3, and Poland) (Figure 7). Respondents in cluster 2 (top right quarter of the biplot) agreed that COVID-19 policies had an impact on the activities of ASF surveillance in their country and that their institutions/working groups are not better prepared to face a similar crisis. Cluster 3 (bottom right quarter of the biplot) shows respondents who mostly gave “neutral” answers (Supplementary Material 9). Perceptions of respondents from the same country can diverge, as illustrated by answers given by respondents from Austria and Belgium (Figure 7).

#### Respondents' Opinion on Potential Areas of Improvement to Be Considered by Veterinary Authorities Planning for Future Crises

**Figure 8:**
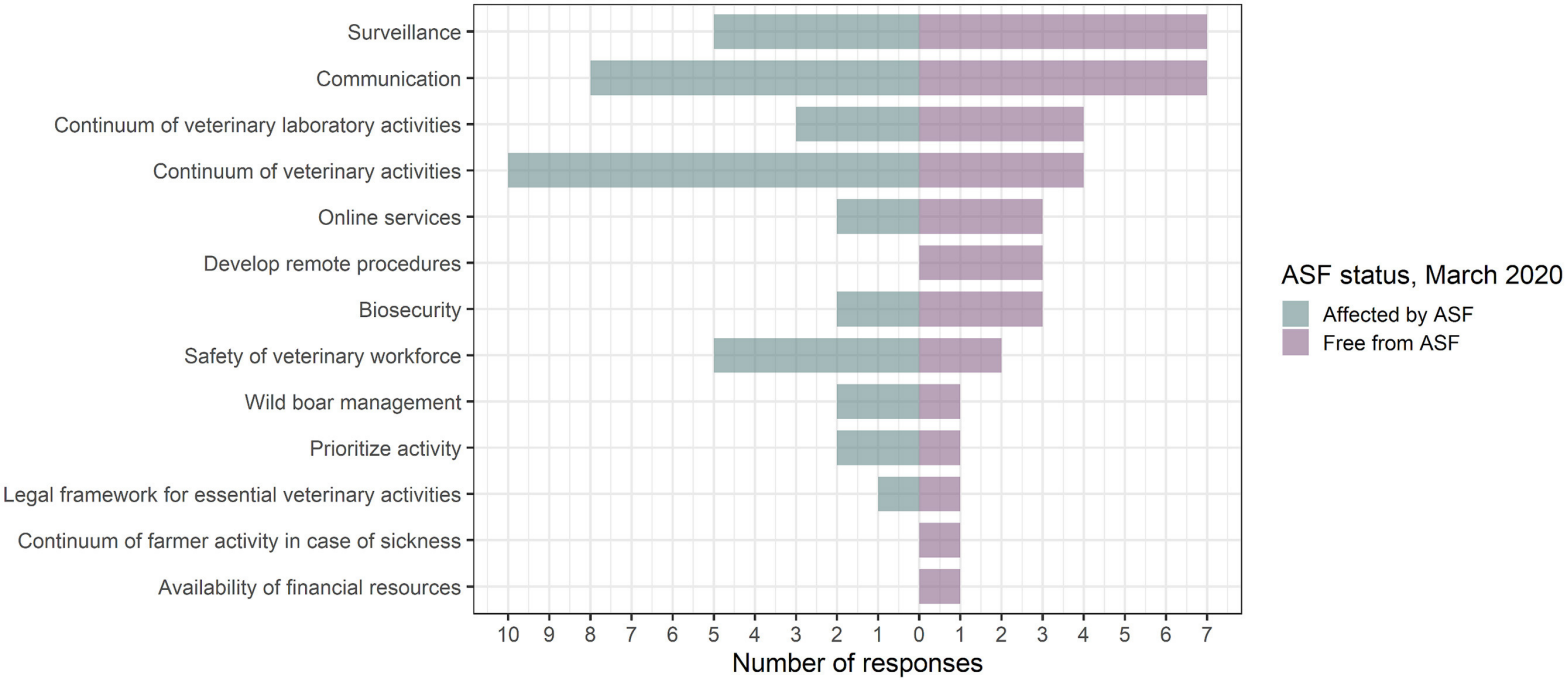
Themes identified in 25 respondents' answers regarding potential areas of improvement for the veterinary authorities to be better prepared for future crisis (two respondents did not answer).

In this section, the respondents used their own words to answer, in three points, to an open-ended question on potential areas of improvement to be considered by the veterinary authorities during lockdown-like situations. Responses were provided in English. In total, 78 answers were analyzed (one respondent skipped answer; one answered “I don't know” to the three points and was not included in the analysis; three respondents mentioned more than one area of improvement per point and all were included in the analysis). Figure 8 presents the themes/concepts mentioned by the respondents in their responses that emerged from the thematic analysis. Supplementary Material 10 shows, for each theme, the different subthemes mentioned. Answers were characterized by their diversity. Respondents pointed out communication (15 mentions, 19.2%) as the major area of improvement for veterinary authorities (e.g., intra- and inter-organization exchanges but also communication with stakeholders, field workers, the public, and hunters). Respondents also called attention on the importance of raising awareness on ongoing epidemics, such as ASF, while another sanitary crisis is ongoing. Respondents identified the continuum of veterinary activities (e.g., mobility and mobilization of the veterinary workforce, supply of equipment, and maintaining minimum veterinary activities) as the second priority area (14 mentions, 17.9%) on which veterinary authorities should focus in the event of a similar crisis. They pinpointed surveillance (active and passive) as the third priority area (12 mentions, 15.4%). We observed differences in the respondents' answers depending on the ASF status of their country (i.e., affected, *n* = 40 answers analyzed, or free from ASF, *n* = 38). Mainly, respondents from countries affected by ASF prioritized the continuum of veterinary activities over other measures during crisis (10/40 mentions, 25%, vs. 4/38, 10.5%, in countries free from ASF) while respondents from countries free from ASF emphasized the importance of surveillance activities (7/38, 18.4%, vs. 5/40, 12.5%, in affected countries) (Figure 8).

## Discussion

Disruption in animal health services and in surveillance activities of zoonoses and TADs can have a severe impact on animal and human health, animal welfare, food security (55, 56), and the economy (57). Our study contributes to a better understanding of the situation faced by the European veterinary services during the first year of the COVID-19 crisis and provides information on how animal health professionals involved in the surveillance and control of ASF adapted their routine tasks to an unprecedented crisis. In particular, it completes the data obtained from the survey conducted among CVOs by the Food and Agriculture Organization of the United Nations (FAO) in July 2020 to evaluate the impact of the COVID-19 on the delivery of state and private veterinary services, which did not receive any answer from European countries (56). By presenting the sanitary situation and economic context in the 24 responding countries over the study period, this paper contextualizes the responses to the questionnaire. Our findings suggest that, in term of crisis management, context matters. Our study confirmed that the pandemic has sometimes shifted priorities of the professionals engaged in the surveillance and control of ASF, trying to reach an acceptable balance between maintaining essential veterinary services and guaranteeing the personal biosafety and biosecurity of the involved personnel, in an extremely instable context, in which knowledge on COVID-19 was not as extensive as today (58). We evidenced that veterinary authorities, institutions, and stakeholders involved in ASF-related activities showed resilience over the period March-May 2020 and successfully adapted the ASF surveillance and control effort.

Our study revealed country-specific variations in the crisis management. Overall, the national crisis management strategies in the answering countries largely followed the EFSA's recommendations on control measures to stop the spread of ASF in wild boars (38). In particular, the maintenance of passive and active ASF surveillance activities in the wild boar population, including active search for carcasses, was given a high priority. Furthermore, despite the implementation of stay-at-home orders in most European countries (2), hunting activities were sustained (sometimes partially) over the study period in three fourth of the responding countries, which could have helped mitigating the intensity of wild boar-domestic pig contacts and therefore the risk of incursion of ASF in the domestic pig population (55). Biosecurity measures to prevent the introduction of ASF in non-affected areas, such as border controls, and communication campaign (mostly online) to increase awareness of hunters, travelers, and commuters, among other, were maintained in most responding countries. Border closure was even mentioned as having a positive impact on ASF control and surveillance activities. Indeed, by reducing flows of people and goods, border closure scaled down the number of controls to be performed at the border and thereby the risk of ASF introduction event (59).

In several responding countries, activities of veterinary practitioners on pig farms were reduced to essential visits only. Limiting the professional monitoring of animal health status, including routine health checks, treatments, vaccines, and diagnostics, hampers the timely detection of and delays intervention against potential health issues, ultimately affecting the health and welfare of farmed animals (60). Respondents cited the continuum of veterinary services and surveillance activities as the second and third top activities, respectively, to be maintained during a crisis. Notably, respondents raised the importance of securing the personal biosafety and biosecurity of the field actors. This further highlights the crucial importance of recognizing animal health services as essential activities during crisis (61). In particular, adequate workforce capacity and mobility as well as equipment supply must be guaranteed to the professionals involved in animal health, food safety, and surveillance and control of animal epidemics. Especially, shortage in PPE can affects negatively on-farm biosecurity (38, 62, 63) and prevention of zoonotic diseases (64, 65).

We identified communication as the first area of improvement for the veterinary authorities in times of crisis. Importantly, in a period where most people were working remotely, some respondents recognized the need for better online communication tools and IT infrastructure/facilities. The importance of planning crisis-adapted protocols was also reported; in particular, respondents brought up the need for protocols allowing remote inspection and control procedures. This advocates for national preparedness plans to include technical preparedness. Indeed, to tackle inter-, intra-, and extra-institution challenges in communication and to make remote official procedures possible, IT tools and communication strategies must be developed prior crisis and the personnel must be familiarized with them.

Our approach presents several limitations. First, the response rate to the questionnaire was low (46% of the targeted countries), although higher than the response rate to the survey conducted by the FAO (30/187, 16%) (56). Possible reasons for non-response in our sample might include a lack of interest in the survey topic, lack of time due to COVID-19-related disruption in daily personal and working life, length of the survey (66), and “survey fatigue” resulting from the rise in survey requests during the COVID-19 pandemic and inducing decreased response rates (67). Moreover, to avoid overwhelming potential respondents, we only sent one reminder. Sending a second reminder, extending the period to complete the survey, or using traditional modes of data collection (i.e., not Web-based) might have increased the response rate, although this seems unlikely, as demonstrated in Smith et al. (68) and van Gelder et al. (69). Second, whether or not the responses for each country are representative of the country of interest is open to debate. The questionnaire did not target a specific group of respondents, rather a large audience, in order to include responses from professionals with different backgrounds and get a broad overview of the situation in each country. Unfortunately, with the exception of Austria and Belgium, we obtained only one completed questionnaire per country, in most cases completed by a veterinary officer. Nevertheless, we assume that veterinary officers have a good knowledge of the situation in their country to provide representative answers, including information on the management of wild boar populations or the challenges faced by laboratories. Interestingly, Slovenia sent back one questionnaire that was completed by a multidisciplinary team of professionals engaged in ASF-related activities; however, all respondents were not listed and could not be characterized. Third, survey participants from the same country have, in some occasions, provided dissimilar answers to questions related to national strategy while we received a relatively important proportion of answers “I don't know” or “Neutral.” Discrepancies in the answers of respondents from the same countries and lack of awareness on the national strategy might indicate shortcomings in vertical (top-down) and horizontal (inter-institutional) communication or be attributed to differences in the professional field of expertise and activity. Finally, the ASF status in the countries (affected vs. free from ASF) may have affected the responses such as evidenced in the results obtained from the sixth section of the questionnaire (potential areas of improvement), although this pattern could not be generalized to other answers, probably due to the diversity of the answers and the modest sample size (e.g., the variable “ASF status” presented a negligible contribution to different dimensions of the MCA, results not shown).

The pandemic has lasted much longer than anticipated and such crisis may become more frequent in the future (70, 71). If short-term measures to tackle the immediate impacts of the COVID-19 PHSMs on ASF-related activities worked relatively well in most European countries and their Eastern neighbors, the long-term implications of the pandemic and its impacts on the surveillance and management of ASF remain largely unclear (55). Only a small majority of the survey participants considered their institution/working group as better prepared to face a similar crisis. This finding advocates the importance of information and experience sharing during and after the crisis. Lessons learnt from the COVID-19 pandemic should help building country-specific sustainable strategies for efficiently maintaining veterinary activities when lockdown policies are in place. Within a holistic One Health approach, preparedness and response plan to disruptive global crises must anticipate syndemic situations (72) to ensure and strengthen the surveillance of TADs (73).

## Data Availability Statement

The anonymized dataset generated and analyzed for this study as well as the R codes used are publicly available on figshare at: https://doi.org/10.6084/m9.figshare.16702474.v1.

## Ethics Statement

Ethical review and approval was not required for the study on human participants in accordance with the local legislation and institutional requirements. The patients/participants provided their written informed consent to participate in this study.

## Author Contributions

AD-L and AK developed and tested the questionnaire, wrote, reviewed, and edited the manuscript. AD-L retrieved email addresses and sent the questionnaire, analyzed the data in R and produced the figures, and wrote the original draft. Both authors provided critical feedback and helped shape the message.

## Conflict of Interest

The authors declare that the research was conducted in the absence of any commercial or financial relationships that could be construed as a potential conflict of interest.

## Publisher's Note

All claims expressed in this article are solely those of the authors and do not necessarily represent those of their affiliated organizations, or those of the publisher, the editors and the reviewers. Any product that may be evaluated in this article, or claim that may be made by its manufacturer, is not guaranteed or endorsed by the publisher.

## Supplementary Material

Supplementary Material 1 PDF version of the Google Forms of the questionnaire sent for this study.

Supplementary Material 2 Sanitary situation and economic context in the 24 responding countries over the study period, March-May 2020. **(A)** Mean wild boar density per country [computed from (35)]; **(B)** Mean domestic pig density per country calculated using the data extracted from the Gridded Livestock of the World (GLW 3) database (https://dataverse.harvard.edu/dataverse/glw_3); **(C)** COVID-19 confirmed deaths per million from March to May 2020 calculated using data retrieved from Our World in Data (37); **(D)** Gross domestic product per capita (37).

Supplementary Material 3 Results of the principal component analysis (PCA). **(A)** Scree plot; **(B)** Correlation circle (colors show the contribution to each variable to first and second dimension of the PCA); **(C)** Contribution of the variables to first (Dim1), second (Dim2), and third (Dim3) dimension of the PCA.

Supplementary Material 4 Countries' clusters obtained using the hierarchical clustering on principal components (HCPC) algorithm on the first three dimensions of the principal component analysis (PCA). Clusters described countries through seven indicators of the sanitary situation and economic context over the study period March-May 2020. The column “v.test” represents the statistical value used to determine the significance (“p.value”) of the variables describing the group (a positive value indicates an over-representation of the modality under consideration; a negative value represents an under-representation); the column “Mean in category” (“sd in category”) represents the mean (sd) of the variable in the cluster; the column “Overall mean” (“Overall sd”) represents the mean (sd) of the variable in the dataset.

Supplementary Material 5 Descriptive summary of the answers to the questionnaire: section two, hunting and wild boar management activities.

Supplementary Material 6 Descriptive summary of the answers to the questionnaire: sections three and four: activity of the veterinary practitioners, laboratory activities, ASF training, ASF surveillance and control activities.

Supplementary Material 7 Results of the multiple correspondence analysis (MCA). **(A)** Scree plot; **(B)** Variables plotted according to their squared loadings (correlation between variables and MCA principal dimensions).

Supplementary Material 8 Contribution of the variables to first (Dim1), second (Dim2), third (Dim3), and fourth (Dim4) dimension of the multiple correspondence analysis (MCA).

Supplementary Material 9 Respondents' clusters obtained using the hierarchical clustering on principal components (HCPC) algorithm on the first four dimensions of the multiple correspondence analysis (MCA). Clusters group respondents using their responses to the Likert scale items describing their perception of the impact of the COVID-19 PHSMs on the surveillance and control of ASF in their countries. The column “Cla/Mod” indicates what percentage of all individuals with this modality are in this cluster. The column “Mod/Cla” indicates what percentage of all individuals in the cluster have this modality. The column “Global” indicates the percentage of subjects out of the total population with this modality. The column “v.test” represents the statistical value used to determine the significance of the variables describing the group (a positive value indicates an over-representation of the modality under consideration; a negative value represents an under-representation).

Supplementary Material 10 Hierarchical tree summarizing the themes and subthemes identified through thematic analysis of the answer to the open-ended question (section six) “*In your opinion, what are the three main points the Veterinary Authorities should focus on in the event of another Covid-19 lockdown (or any other crisis that would shut down the country)*?”.

## References

[1] World Health Organization. WHO Director-General's Opening Remarks at the Media Briefing on Covid-19-−11 March 2020. (2020). Available online at: https://www.who.int/director-general/speeches/detail/who-director-general-s-opening-remarks-at-the-media-briefing-on-covid-19-−11-march-2020 (accessed March 29, 2021).

[2] Desvars-Larrive A Dervic E Haug N Niederkrotenthaler T Chen J Di Natale A. A structured open dataset of government interventions in response to Covid-19. ?Sci Data. (2020) 7:285. 10.1038/s41597-020-00609-9 32855430 PMC7452888

[3] Hale T Angrist N Goldszmidt R Kira B Petherick A Phillips T. A global panel database of pandemic policies (Oxford Covid-19 government response tracker). Nat Hum Behav. (2021) 5:529–38. 10.1038/s41562-021-01079-8 33686204

[4] World Health Organization. Coronavirus Disease (Covid-19): Herd Immunity, Lockdowns and Covid-19. (2020). Available online at: https://www.who.int/news-room/q-a-detail/herd-immunity-lockdowns-and-covid-19 (accessed March 29, 2021).

[5] Vardoulakis S Sheel M Lal A Gray D. Covid-19 environmental transmission and preventive public health measures. Aust N Z J Public Health. (2020) 44:333–5. 10.1111/1753-6405.13033 32833313 PMC7461436

[6] Patrício Silva AL Prata JC Walker TR Duarte AC Ouyang W Barcelò D. Increased plastic pollution due to Covid-19 pandemic: challenges and recommendations. Chem Eng J. (2021) 405:126683. 10.1016/j.cej.2020.126683 32834764 PMC7430241

[7] Lambert H Gupte J Fletcher H Hammond L Lowe N Pelling M. Covid-19 as a global challenge: towards an inclusive and sustainable future. Lancet Planet Health. (2020) 4:e312–4. 10.1016/S2542-5196(20)30168-6 32702296

[8] The Lancet Infectious D. The intersection of Covid-19 and mental health. Lancet Infect Dis. (2020) 20:1217. 10.1016/S1473-3099(20)30797-0 33038942 PMC7544473

[9] Meyer M Bindelglas E Kupferman ME Eggermont AM. The ongoing Covid-19 pandemic will create a disease surge among cancer patients. Ecancermedicalscience. (2020) 14:ed105. 10.3332/ecancer.2020.ed105 33082857 PMC7532024

[10] Richards M Anderson M Carter P Ebert BL Mossialos E. The impact of the Covid-19 pandemic on cancer care. Nat Cancer. (2020) 1:565–7. 10.1038/s43018-020-0074-y 35121972 PMC7238956

[11] Cronin A Railey S Fortune D Wegener D Davis J. Notes from the field: effects of the Covid-19 response on tuberculosis prevention and control efforts—United States, March–April 2020. Morb Mortal Wkly Rep. (2020) 69:971–2. 10.15585/mmwr.mm6929a4 32701944 PMC7377818

[12] Hamilton W. Cancer diagnostic delay in the Covid-19 era: what happens next? Lancet Oncol. (2020) 21:1000–2. 10.1016/S1470-2045(20)30391-0 32702312 PMC7834491

[13] Lazzerini M Barbi E Apicella A Marchetti F Cardinale F Trobia G. Delayed access or provision of care in Italy resulting from fear of Covid-19. Lancet Child Adolesc Health. (2020) 4:e10–1. 10.1016/S2352-4642(20)30108-5 32278365 PMC7146704

[14] Middleton J Reintjes R Lopes H. Meat plants—a new front line in the Covid-19 pandemic. BMJ. (2020) 370:m2716. 10.1136/bmj.m2716 32646892

[15] Taylor CA Boulos C Almond D. Livestock plants and Covid-19 transmission. Proc Natl Acad Sci U S A. (2020) 117:31706–15. 10.1073/pnas.2010115117 33214147 PMC7749337

[16] Stewart A Kottasová I Khaliq A. Why meat processing plants have become Covid-19 hotbeds. CNN. (2020) 27 June 2020.

[17] Ross A. Coronavirus outbreaks and closures hit Europe's meat factories. Unearthed. (2020) 24 June 2020. Available online at: https://unearthed.greenpeace.org/2020/06/24/coronavirus-outbreaks-and-closures-hit-europes-meat-factories/

[18] Marchant-Forde JN Boyle LA. Covid-19 effects on livestock production: a one welfare issue. Front Vet Sci. (2020) 7:585787. 10.3389/fvets.2020.585787 33195613 PMC7554581

[19] Felix I Martin A Mehta V Mueller C. US Food Supply Chain: Disruptions Implications From Covid-19. (2020). Available online at: https://www.mckinsey.com/industries/consumer-packaged-goods/our-insights/us-food-supply-chain-disruptions-and-implications-from-covid-19 (accessed April 1, 2020).

[20] European Commission. Short-Term Outlook for Eu Agricultural Markets in 2020. DG Agriculture and Rural Development (2020).

[21] United States Department of Agriculture—Foreign Agricultural Service. Global Agricultural Information Network. Poultry and Products (2020).

[22] World Organisation for Animal Health. Responding to the Covid-19 Crisis: The Contribution of the Veterinary Profession. (2020). Available online at: https://www.oie.int/en/for-the-media/press-releases/detail/article/responding-to-the-covid-19-crisis-the-contribution-of-the-veterinary-profession/ (accessed March 29, 2021).

[23] World Health Organization. Shortage of Personal Protective Equipment Endangering Health Workers Worldwide. (2020). Available online at: https://www.who.int/news/item/03-03-2020-shortage-of-personal-protective-equipment-endangering-health-workers-worldwide (accessed March 30, 2021).

[24] European Centre for Disease Prevention and Control. Coronavirus Disease 2019 (Covid-19) Pandemic: Increased Transmission in the EU/EEA and the UK–seventh update. Stockholm: ECDC (2020).

[25] World Health Organisation for Animal Health. Responding to the Covid-19 Crisis: The Contribution of the Veterinary Profession. (2020). Available online at: https://www.oie.int/en/for-the-media/press-releases/detail/article/responding-to-the-covid-19-crisis-the-contribution-of-the-veterinary-profession/ (accessed April 1, 2021).

[26] World Organisation for Animal Health. In Italy, Multidisciplinary Collaboration Has Been Crucial to Address Covid-19. (2020). Available online at: https://rr-europe.oie.int/en/news/address-covid-19-with-support-from-italy/ (accessed April 1, 2021).

[27] World Organisation for Animal Health. Spanish Animal Health Laboratories Help Break Covid-19 Transmission Chains in Humans. (2020). Available online at: https://rr-europe.oie.int/en/news/spain-helps-break-covid-19-transmission-chains/ (accessed April 1, 2021).

[28] World Organisation for Animal Health. Covid-19 and Veterinary Activities Designated as Essential. (2020). Available online at: https://www.oie.int/en/for-the-media/press-releases/detail/article/covid-19-and-veterinary-activities-designated-as-essential/ (accessed December 20, 2020).

[29] Communication from the Commission on the Implementation of the Green Lanes under the Guidelines for Border Management Measures to Protect Health Ensure the Availability of Goods Essential Services (2020/C 96 I/01). OJEU. (2020) 63. Available online at: https://eur-lex.europa.eu/legal-content/EN/ALL/?uri=CELEX%3A52020XC0324%2801%29

[30] Council Directive 2002/60/EC of 27 June 2002 Laying Down Specific Provisions for the Control of African Swine Fever and Amending Directive 92/119/EEC as Regards Teschen Disease and African Swine Fever (2002).

[31] World Organisation for Animal Health. Terrestrial Animal Health Code. World Organization for Animal Health (2019)

[32] Cwynar P Stojkov J Wlazlak K. African swine fever status in Europe. Viruses. (2019) 11:310. 10.3390/v11040310 30935026 PMC6521326

[33] Sánchez-Cordón PJ Montoya M Reis AL Dixon LK. African swine fever: a re-emerging viral disease threatening the global pig industry. Vet J. (2018) 233:41–8. 10.1016/j.tvjl.2017.12.025 29486878 PMC5844645

[34] Torresi C Fiori M Bertolotti L Floris M Colitti B Giammarioli M. The evolution of African swine fever virus in Sardinia (1978–2014) as revealed by whole-genome sequencing and comparative analysis. Transbound Emerg Dis. (2020) 67:1971–80. 10.1111/tbed.13540 32163673

[35] Rolesu S Mandas D Loi F Oggiano A Dei Giudici S Franzoni G. African swine fever in smallholder sardinian farms: last 10 years of network transmission reconstruction and analysis. Front Vet Sci. (2021) 8:692448. 10.3389/fvets.2021.692448 34395576 PMC8361751

[36] Rowlands RJ Michaud V Heath L Hutchings G Oura C Vosloo W. African swine fever virus isolate, Georgia, 2007. Emerg Infect Dis. (2008) 14:1870–4. 10.3201/eid1412.080591 19046509 PMC2634662

[37] Sauter-Louis C Forth JH Probst C Staubach C Hlinak A Rudovsky A. Joining the club: first detection of African swine fever in wild boar in Germany. Transbound Emerg Dis. (2020) 68:1744–52. 10.1111/tbed.13890 33085828

[38] European Food Safety Authority (EFSA) Boklund A Bøtner A Chesnoiu VT Depner K Desmecht D. Epidemiological analyses of African swine fever in the European union (November 2018 to October 2019). EFSA. (2019) 77–81. 10.2903/j.efsa.2020.5996 PMC7009685 32625771

[39] Regulation Regulation (EU) 2016/679 of the European Parliament and of the Council of 27 April 2016 on the protection of natural persons with regard to the processing of personal data and on the free movement of such data and repealing directive 95/46/EC (General Data Protection Regulation) (Text with EEA Relevance) 32016R0679 (2016) 59:1. Available online at: https://eur-lex.europa.eu/eli/reg/2016/679/oj

[40] Pittiglio C Khomenko S Beltran-Alcrudo D. Wild boar mapping using population-density statistics: from polygons to high resolution raster maps. PLoS ONE. (2018) 13:e0193295. 10.1371/journal.pone.0193295 29768413 PMC5955487

[41] Gilbert M Nicolas G Cinardi G Van Boeckel TP Vanwambeke SO Wint GRW. Global distribution data for cattle, buffaloes, horses, sheep, goats, pigs, chickens and ducks in 2010. ?Sci Data. (2018) 5:180227. 10.1038/sdata.2018.227 30375994 PMC6207061

[42] GitHub. Data on Covid-19 (Coronavirus) by Our World in Data. (2020). Available online at: https://github.com/owid/covid-19-data/tree/master/public/data (accessed March 15, 2021).

[43] RStudio Team. Rstudio: Integrated Development for R. Boston: RStudio (2020).

[44] R Core Team. R: A Language and Environment for Statistical Computing. Vienna: R Foundation for Statistical Computing (2021).

[45] Lever J Krzywinski M Altman N. Principal component analysis. Nat Methods. (2017) 14:641–2. 10.1038/nmeth.4346

[46] Kassambara A. Practical Guide to Principal Component Methods in R. Wrocław: CreateSpace Independent Publishing Platform (2017). p. 170.

[47] Sullivan GM Artino AR. Jr. Analyzing and Interpreting Data from Likert-Type Scales. J Grad Med Educ. (2013) 5:541–2. 10.4300/JGME-5-4-18 24454995 PMC3886444

[48] Jamieson S. Likert scales: how to (ab)use them. Med Educ. (2004) 38:1217–8. 10.1111/j.1365-2929.2004.02012.x 15566531

[49] Lê S Josse J Husson F. Factominer: a package for multivariate analysis. J Stat Softw. (2008) 25:1–18. 10.18637/jss.v025.i01

[50] Kassambara A Mundt F. Factoextra: extract and visualize the results of multivariate data analyses. R Package Version 107. (2020). https://CRAN.R-project.org/package=factoextra

[51] Josse J Husson F. >Missmda: A package for handling missing values in multivariate data analysis. J Stat Softw. (2016) 70:1–31. 10.18637/jss.v070.i01

[52] Braun V Clarke V. Successful Qualitative Research: A Practical Guide for Beginners. London: SAGE (2013).

[53] Wickham H. Ggplot2: Elegant Graphics for Data Analysis. New York, NY: Springer-Verlag (2016). 10.1007/978-3-319-24277-4_9

[54] Bryer J Speerschneider K. Likert: Analysis Visualization Likert Items. R Package Version 1.3.5. (2016). Available online at: https://CRAN.R-project.org/package=likert

[55] Gortázar C. de la Fuente J. Covid-19 Is Likely to Impact Animal Health. Prev *Vet Med*. (2020) 180:105030. 10.1016/j.prevetmed.2020.105030 32447153 PMC7255270

[56] Food and Agriculture Organization of the United Nations. Impact of Covid-19 on the Delivery of Veterinary Services and Animal Disease Reporting. Rome: FAO (2021).

[57] Guan D Wang D Hallegatte S Davis SJ Huo J Li S. Global supply-chain effects of Covid-19 control measures. Nat Hum Behav. (2020) 4:577–87. 10.1038/s41562-020-0896-8 32493967

[58] Covid research: a year of scientific milestones. Nature. (2021) 10.1038/d41586-020-00502-w 32221507

[59] Sugiura K Kure K Kato T Kyutoku F Haga T. Change in the ASF entry risk into Japan as a result of the Covid-19 pandemic. Transbound Emerg Dis. (2021) 68:1700–3. 10.1111/tbed.13836 32969591

[60] Hashem NM González-Bulnes A Rodriguez-Morales AJ. Animal welfare and livestock supply chain sustainability under the Covid-19 outbreak: an overview. Front Vet Sci. (2020) 7:582528. 10.3389/fvets.2020.582528 33195601 PMC7593325

[61] World Organisation for Animal Health (OIE) World Veterinary Association (WVA). Covid-19 and Veterinary Activities Designated as Essential. OIE/WVA Joint Statement. (2020). Available online at: https://www.oie.int/en/covid-19-and-veterinary-activities-designated-as-essential/

[62] Kim Y Yang M Goyal SM Cheeran MCJ Torremorell M. Evaluation of biosecurity measures to prevent indirect transmission of porcine epidemic diarrhea virus. BMC Vet Res. (2017) 13:89. 10.1186/s12917-017-1017-4 28381304 PMC5382501

[63] Bellini S Rutili D Guberti V. Preventive measures aimed at minimizing the risk of African swine fever virus spread in pig farming systems. Acta Vet Scand. (2016) 58:82. 10.1186/s13028-016-0264-x 27899125 PMC5129245

[64] Brown VR Bowen RA Bosco-Lauth AM. Zoonotic pathogens from feral swine that pose a significant threat to public health. Transbound Emerg Dis. (2018) 65:649–59. 10.1111/tbed.12820 29388363

[65] Dignard C Leibler JH. Recent research on occupational animal exposures and health risks: a narrative review. Curr Environ Health Rep. (2019) 6:236–46. 10.1007/s40572-019-00253-5 31823248 PMC7099379

[66] Fan W Yan Z. Factors affecting response rates of the web survey: a systematic review. Comput Hum Behav. (2010) 26:132–9. 10.1016/j.chb.2009.10.015

[67] de Koning R Egiz A Kotecha J Ciuculete AC Ooi SZY Bankole NDA. Survey fatigue during the Covid-19 pandemic: an analysis of neurosurgery survey response rates. Front Surg. (2021) 8:690680. 10.3389/fsurg.2021.690680 34458314 PMC8388838

[68] Smith MG Witte M Rocha S Basner M. Effectiveness of incentives and follow-up on increasing survey response rates and participation in field studies. BMC Med Res Methodol. (2019) 19:230. 10.1186/s12874-019-0868-8 31805869 PMC6896692

[69] van Gelder MM Bretveld RW Roeleveld N. Web-based questionnaires: the future in epidemiology? Am J Epidemiol. (2010) 172:1292–8. 10.1093/aje/kwq291 20880962

[70] Telenti A Arvin A Corey L Corti D Diamond MS García-Sastre A. After the pandemic: perspectives on the future trajectory of Covid-19. Nature. (2021) 596:495–504. 10.1038/s41586-021-03792-w 34237771

[71] The Lancet Planetary H. A pandemic era. Lancet Planet Health. (2021) 5:e1. 10.1016/S2542-5196(20)30305-3 33421401 PMC7833825

[72] Fronteira I Sidat M Magalhães JP de Barros FPC Delgado AP Correia T. The Sars-Cov-2 pandemic: a syndemic perspective. One *Health*. (2021) 12:100228. 10.1016/j.onehlt.2021.100228 33614885 PMC7887445

[73] Nkengasong JN. Covid-19: unprecedented but expected. Nat Med. (2021) 27:364. 10.1038/s41591-021-01269-x 33723449

