# Supplementary material for: Surveillance and Control of African Swine Fever in the Early Phase of the COVID-19 Pandemic, March-May 2020: A Multi-Country E-Survey
Source: Front Vet Sci. 2022 Jun 6;9:867631. doi: 10.3389/fvets.2022.867631 (PMC9238323; doi:10.3389/fvets.2022.867631)
Supplement: Supplementary Material 1 — PDF version of the Google Forms of the questionnaire sent for this study. [file Data_Sheet_1.zip › Supplementary Material 1.PDF]

Surveillance and control of African swine fever in Europe during the Covid-19 health crisis

*Required

Background

A report from EFSA mentions that the EU Veterinary Services and other Stakeholders involved in pig production and wild boar management identified “Surveillance” as one of the most significant knowledge gaps in the prevention and control of African swine fever. In particular, the following aspects were perceived as important:
(1) Sensitivity of border inspection controls to reduce the risk of introduction of African swine fever virus;
(2) Methods for passive surveillance to improve early detection (i.e. methods for improved detection of wild boar carcasses); and
(3) Sampling protocols and diagnostics (e.g. improved sensitivity of the tests and the development of rapid diagnostic tests able to be performed in the field) [1].

In March-May 2020, most European countries were under lockdown or experiencing lockdown-like measures, and were therefore facing unprecedented restrictions on individual movements and global activity. How did European Veterinary Services and other Stakeholders involved in pig production and wild boar management face this challenging time? How did they adapt and implement surveillance and control measures of African swine fever while the countries were shut down and the entire world focused on another viral disease?

We aim to assess to which extend Covid-19 lockdown (or lockdown-like measures) has affected:
i) The surveillance and control of African swine fever in the different European countries (e.g. border controls, search for wild board carcasses, timeliness of laboratory diagnosis),
ii) The response of the Veterinary Authorities to African swine fever outbreaks.

[1] European Food Safety Authority, Álvarez J, Bicout D, Boklund A, Bøtner A, et al. (2019) Research gap analysis on African swine fever. EFSA Journal, 17 (8): e05811. doi: 10.2903/j.efsa.2019.5811.

Purpose of the questionnaire

This questionnaire aims to collect qualitative data on the impact of the Covid-19 lockdown (or lockdown-like measures) on the management of African swine fever in European countries.
We also aim to collect qualitative data on potential adaptive measures implemented by the Veterinary Authorities, diagnostic Laboratories, and other Stakeholders as well as how key actors involved in the surveillance and control of African swine fever perceived the epidemiological consequences of the Covid-19 crisis on the spread of African swine fever in their respective countries.

We choose to study the period March 2020 (first national lockdown in place) - May 2020 (most countries have re-opened borders).

Number of questions: 24
Estimated time to fill-up the questionnaire: 30 min.

Contact person

This research is conducted by the University of Veterinary Medicine Vienna, Austria.

Contact person: Ass.-Prof. Amélie Desvars-Larrive, DVM, PhD
Unit of Veterinary Public Health and Epidemiology,
University of Veterinary Medicine Vienna, Veterinärpl. 1, 1210 Vienna, Austria

Email: amelie.desvars@vetmeduni.ac.at

I. Information on the Respondent

Privacy policy

The protection of your personal data is of particular concern to us in this survey. Your data will therefore only be collected and processed on the basis of the legal regulations (§ 7 DSG, § 89 GDPR and FOG).

This survey is conducted within the framework of a scientific work / thesis at the University of Veterinary Medicine Vienna (Vetmeduni Vienna). The data can be downloaded by the assessor of the scientific work for the purpose of performance evaluation. The data will not be passed on to third parties. In accordance with Art 89 (1) GDPR, the data collected may in principle be stored without restriction.

There is a right of access by the person(s) responsible for this study to the personal data collected, as well as the right to correct, delete, restrict the processing of the data and a right to object to the processing and the right to data transferability.

If you have any questions regarding this survey, please contact the person responsible for this study: Amélie Desvars-Larrive (amelie.desvars@vetmeduni.ac.at) at Vetmeduni Vienna, Veterinaerplatz 1, 1210 Vienna, Austria.

For fundamental legal questions in connection with the General Data Protection Regulation (GDPR) / Datenschutzgesetz (DSG) and Forschungsorganisationsgesetz (FOG), please contact the Data Protection Officer of the Vetmeduni Vienna (datenschutz@vetmeduni.ac.at). There is also the right to complain to the data protection authority (e.g. via dsb@dsb.gv.at).

By participating in this study, you agree to the processing of your personal data.

1. Respondent´s country *

____

2. Respondent´s first name and family name *

____

3. The Respondent is *

Tick all that apply.

- [ ] A Veterinary Officer
- [ ] The Head of/working in a Diagnostic Laboratory
- [ ] A Member of the EU African swine fever Task Force

Other: [ ] ____________________

4. Respondent´s Institution *

____

5. Respondent´s position in the Institution *

____

6. Respondent´s email address

____

7. Further contact

Tick all that apply.

- [ ] You agree to be contacted in case we need further information to complete the form.
- [ ] You wish to receive the results of our study.

II. Hunting and feral pig management

8. 1. Between March and May 2020, were there restrictions in your country for accessing wooden areas and national parks? *

Mark only one oval.

- Yes
- No
- I don´t know
- Other: ______________________

9. 2. In your country, was hunting allowed between March and May 2020? *

Mark only one oval.

- Yes (the whole period) *Skip to question 12*
- No (it was not allowed the whole period or part of it) *Skip to question 10*
- I don´t know *Skip to question 13*

II. Hunting and feral pig management

10. 2.1. Please, specify the date from which hunting was not permitted

____

Example: 7 January 2019

11. 2.2. Please, specify the date at which hunting was permitted again

____

Example: 7 January 2019

II. Hunting and feral pig management

12. 3. Between March and May 2020, could hunters enter hunting areas in order to maintain feral pig hunting activity to a certain level? *

Mark only one oval.

- Yes
- No
- Yes, but they needed a document exempting them from the stay-at-home rule
- I don´t know

II. Hunting and feral pig management

13. 4. Are there usually winter feeding areas for feral pigs in your country? *

Mark only one oval.

- Yes *Skip to question 14*
- No *Skip to question 17*
- I don´t know *Skip to question 17*

II. Hunting and feral pig management

14. 4.1. Were the winter feeding areas maintained between March and May 2020 in your country? *

Mark only one oval.

- Yes *Skip to question 17*
- No *Skip to question 17*
- Partially *Skip to question 15*
- I don´t know *Skip to question 17*

II. Hunting and feral pig management

15. 4.2. The winter feeding areas were maintained PARTIALLY between March and May 2020: *

Mark only one oval.

- The number of feeding areas was reduced *Skip to question 17*
- Feeding areas not maintained for a certain period of time between March and May 2020 *Skip to question 16*
- Both (reduced and then not maintained anymore) *Skip to question 16*
- I don´t know *Skip to question 17*
- Other: ____________________

II. Hunting and feral pig management

16. 4.3. Feeding areas were not maintained from (dd/mm/yyyy - dd/mm/yyyy)

____

II. Hunting and feral pig management

17. 5. In "normal time", is there any active search for feral pig carcasses organised in your country as a measure against African swine fever?

Mark only one oval.

- Yes *Skip to question 18*
- No *Skip to question 25*
- I don´t know *Skip to question 25*

II. Hunting and feral pig management

18. 6. Was there active feral pig carcass search between March and May 2020 in your country?

Mark only one oval.

- Yes *Skip to question 19*
- No *Skip to question 23*
- I don´t know *Skip to question 23*

II. Hunting and feral pig management

19. 6.1. Could the search for feral pig carcasses be performed AS USUAL (i.e. absolutely no change in the procedure) in March and May 2020 in your country?

Mark only one oval.

- Yes *Skip to question 23*
- No (there were adaptations/exceptional procedures) *Skip to question 20*
- I don´t know *Skip to question 23*

II. Hunting and feral pig management

20. 6.2. How was the search for feral pig carcasses impacted in your country in March-May 2020? For example:

Mark only one oval.

- The number of persons involved in the search of carcasses was reduced *Skip to question 22*
- Permitting documents (exemption from stay-at-home rule) were required to access the wooden areas *Skip to question 21*
- The number of persons involved was reduced AND permitting documents were needed *Skip to question 21*
- I don´t know
- Other: ________________

II. Hunting and feral pig management

21. 6.3. In your country, who received a special permitting document for movements (i.e. exempting from stay-at-home rule) in wooden areas in the framework of African swine fever surveillance and/or management?

Tick all that apply.

- [ ] Official veterinarians
- [ ] Hunters
- [ ] Forestry workers

Other: [ ] ____________________

II. Hunting and feral pig management

22. 6.4. Other adaptive procedures (related to the Covid-19 context) to the search for feral pig carcasses in your country over the period March-May 2020:

____

____

____

____

____

III. Activities of official veterinarians and swine veterinary practitioners

23. 7. During the “lockdown” (or when lockdown-like measures were in place) in March-May 2020, in your country, farm visits by swine veterinarian practitioners were: *

Mark only one oval.

- ( ) Totally cancelled (not conducted, not postponed)
- ( ) Postponed to after lockdown (the total number of official visits per year stayed the same)
- ( ) Reduced (only farms that needed the most veterinary services were visited)
- ( ) Some “visits” were conducted via phone call or other communication tools
- ( ) Conducted as planned
- ( ) I don´t know
- ( ) Other: ____________________

24. 8. Between March and May 2020, the displacements/visits of swine veterinary practitioners to swine holdings (for any reason) required permitting document for movement (exemption from the stay-at-home rule):

Mark only one oval.

- Yes
- No
- I don´t know

IV. African swine fever management & regulations

25. 9. In your country, between March and May 2020, were there official changes in the regulations or new advice regarding African swine fever management (e.g. derogation or adaptation to the contingency plan) taken as a consequence of the Covid-19 situation? *

Mark only one oval.

- Yes *Skip to question 26*
- No *Skip to question 27*
- I don´t know *Skip to question 27*

IV. African swine fever management & regulations

26. 9.1. In your country, between March and May 2020, which change(s) was/were made in the regulations or which specific advice was/were given regarding the management of the African swine fever in order to face the Covid-19 context? *

IV. African swine fever management & regulations

27. 10. In your country, between March and May 2020, were the training procedures related to African swine fever for, e.g., veterinarians, breeders and hunters: *

Mark only one oval.

- Totally cancelled (no date scheduled yet) *Skip to question 30*
- Conducted as scheduled (physical presence of the participant, planned number of attendees) *Skip to question 30*
- Adapted (physical presence, less persons to respect physical distancing) *Skip to question 30*
- Postponed *Skip to question 28*
- Conducted online *Skip to question 29*
- Other: ______________________

IV. African swine fever management & regulations

28. 10.1. Date when they will start again / have started again (month and year are enough)

____

Example: 7 January 2019

IV. African swine fever management & regulations

29. 10.2. Training procedures related to African swine fever in your country were conducted online

Tick all that apply.

- [ ] For official veterinarians
- [ ] For practitioners
- [ ] For pig breeders
- [ ] For hunters

Other: [ ] ______________________

IV. African swine fever management & regulations

30. 11. Between March and May 2020, were African swine fever laboratory diagnostic tests delayed in your country due to Covid-19? *

Mark only one oval.

- ( ) Yes *Skip to question 31*
- ( ) No *Skip to question 32*
- ( ) No test was required during the studied period *Skip to question 32*
- ( ) I don´t know *Skip to question 32*

IV. African swine fever management & regulations

31. 11.1. Which was/were the most important reason(s) for the delay in the diagnostic tests for African swine fever? *

Tick all that apply.

- [ ] Delay in the transport of samples
- [ ] Reduced number of laboratory workers
- [ ] No/reduced supply of reagents or laboratory materials
- [ ] The laboratory was repurposed for Covid-19 tests
- [ ] I don´t know

Other: [ ] ____________________

IV. African swine fever management & regulations

32. 12. Were some rendering plants closed between March and May 2020 in your country? *

Mark only one oval.

- ( ) Yes *Skip to question 33*
- ( ) No *Skip to question 34*
- ( ) I don´t know *Skip to question 34*

IV. African swine fever management & regulations

33. 12.1. How did the closure of rendering plants affect the management of pig and feral pig carcasses in your country?

Mark only one oval.

- ( ) No impact
- ( ) Small impact (e.g. the carcasses were treated by another rendering plant without any delay)
- ( ) Medium impact (e.g. the carcasses were treated by another rendering plant but with delay)
- ( ) High impact (e.g. the carcasses could not be processed)
- ( ) I don´t know
- ( ) Other: ______________________

IV. African swine fever management & regulations

34. 13. In your country, between March and May 2020, border inspection and control to prevent the introduction of African swine fever were: *

Mark only one oval.

- ( ) Performed as usual
- ( ) Performed but less regularly (e.g. workforce repurposed for Covid-19 control)
- ( ) Not performed
- ( ) I don´t know
- ( ) Other: ______________________

IV. African swine fever management & regulations

35. The next three questions to be answered only if your country has reported cases of African swine fever BEFORE March 2020. *

Mark only one oval.

- ( ) Yes my country has reported cases of African swine fever BEFORE March 2020
  Skip to question 36
- ( ) My country has NOT reported cases of African swine fever BEFORE March 2020
  Skip to question 39

IV. African swine fever management & regulations

(Countries which have reported cases of African swine fever before March 2020)

36. 14. Over the period March-May 2020, was the time between a suspicion of African swine fever in a pig farm (e.g. by a practitioner) and the visit of an official veterinarian longer than usual in your country (that could be attributed to the Covid-19 lockdown or lockdown-like measures)?

Mark only one oval.

- No
- Yes, but the impact can be considered as minor
- Yes, and the impact can be considered as major
- There was no suspicion over the studied period
- I don´t know

37. 15. In your country, between March and May 2020, after an official diagnosis of African swine fever in a PIG FARM, was there any delay (compared to the usual timing) in the implementation of the contingency plan for African swine fever (that could be attributed to the Covid-19 lockdown or lockdown-like measures)?

Mark only one oval.

- No
- Yes, but the impact can be considered as minor
- Yes, and the impact can be considered as major
- There was no diagnosis of African swine fever in pig farm over the studied period
- I don´t know

38. 16. In your country, between March and May 2020, after an official diagnosis of African swine fever in a FERAL PIG, was there any delay (compared to the usual timing) in the implementation of the contingency plan for African swine fever (that could be attributed to the Covid-19 lockdown or lockdown-like measures)?

Mark only one oval.

- No
- Yes
- Yes, but the impact can be considered as minor
- Yes, and the impact can be considered as major
- There was no diagnosis of African swine fever in feral pig over the studied period
- I don´t know

V. Opinion survey: Impact of Covid-19 government interventions on the surveillance and spread of African swine fever

1: Strongly disagree ; 2: Disagree; 3: Neutral; 4: Agree; 5: Strongly agree

39. 17. Overall, Covid-19 restrictive (lockdown-like) measures have prevented the implementation of the usual actions in place against African swine fever in your country *

Mark only one oval.

	1	2	3	4	5	
Strongly disagree						Strongly agree

40. 18. Overall, Covid-19 restrictive (lockdown-like) measures had no impact on the surveillance of African swine fever in your country *

Mark only one oval.

	1	2	3	4	5	
Strongly disagree						Strongly agree

41. 19. In your country, the density of feral pigs has increased due to the Covid-19 situation (e.g. because the number of hunted feral pigs was lower than usual) *

Mark only one oval.

	1	2	3	4	5	
Strongly disagree	⬭	⬭	⬭	⬭	⬭	Strongly agree

42. 20. The border restrictions due to Covid-19 have decreased the risk of introduction of African swine fever in your country (e.g. less incoming people and traffic)

Mark only one oval.

	1	2	3	4	5	
Strongly disagree	⬭	⬭	⬭	⬭	⬭	Strongly agree

43. 21. After the first Covid-19 lockdown, your Institution/Working Group was/is better prepared to ensure its tasks related to the surveillance of African swine fever in the event of subsequent Covid-19 lockdown(s) or any other health crisis *

Mark only one oval.

	1	2	3	4	5	
Strongly disagree	⬭	⬭	⬭	⬭	⬭	Strongly agree

V. Opinion survey: Impact of Covid-19 government interventions on the surveillance and spread of African swine fever

44. The next two statements to be answered only if your country recorded cases of African swine fever before March 2020 *

Mark only one oval.

- ( ) Yes my country has reported cases of African swine fever BEFORE March 2020
  Skip to question 45
- ( ) My country has NOT reported cases of African swine fever BEFORE March 2020
  Skip to question 47

V. Opinion survey: Impact of Covid-19 government interventions on the surveillance and spread of African swine fever

(Countries which have reported cases of African swine fever before March 2020)

45. 22. The reduction in the usual (routine) feral pig management and surveillance activities has favoured the spread of the of the African swine fever virus in the feral pigs population in your country

Mark only one oval.

	1	2	3	4	5	
Strongly disagree	( )	( )	( )	( )	( )	Strongly agree

46. 23. The number of African swine fever cases in domestic pigs increased in your country due to the Covid-19 health crisis

Mark only one oval.

	1	2	3	4	5	
Strongly disagree	( )	( )	( )	( )	( )	Strongly agree

V. Opinion survey: Impact of Covid-19 government interventions on the surveillance and spread of African swine fever

24. In your opinion, what are the three main points the Veterinary Authorities should focus on in the event of another Covid-19 lockdown (or any other crisis that would shut down the country)

The question refers to any other crisis that would lead to lockdown-like measures or would restrict the normal activities of the citizens and institutions.

47. Point 1 *

48. Point 2 *

49. Point 3 *

This content is neither created nor endorsed by Google.

Google Forms
