# Supplementary material for: Surveillance and Control of African Swine Fever in the Early Phase of the COVID-19 Pandemic, March-May 2020: A Multi-Country E-Survey
Source: Front Vet Sci. 2022 Jun 6;9:867631. doi: 10.3389/fvets.2022.867631 (PMC9238323; doi:10.3389/fvets.2022.867631)
Supplement: Supplementary Material 1 — PDF version of the Google Forms of the questionnaire sent for this study. [file Data_Sheet_1.zip › Supplementary Material 4.DOCX]

Supplementary Material 4

**Countries´ clusters obtained using the hierarchical clustering on principal components (HCPC) algorithm on the first three dimensions of the principal component analysis (PCA).** Clusters described countries through seven indicators of the sanitary situation and economic context over the study period March-May 2020. The column “v.test” represents the statistical value used to determine the significance (“p.value”) of the variables describing the group (a positive value indicates an over-representation of the modality under consideration; a negative value represents an under-representation); the column “Mean in category” (“sd in category”) represents the mean (sd) of the variable in the cluster; the column “Overall mean” (“Overall sd”) represents the mean (sd) of the variable in the dataset.

| **Variable** | **v.test** | **Mean in category** | **Overall mean** | **sd in category** | **Overall sd** | **p.value** |
| --- | --- | --- | --- | --- | --- | --- |
| **Cluster 1: ROU** | | | | | | |
| sumCases_PIG | 4.79 | 7366 | 323.33 | 0 | 1470.6 | 1.7e-06 |
| **Cluster 2: HUN** | | | | | | |
| sumCases_WB | 4.28 | 2987 | 205.42 | 0 | 650.02 | 1.88e-05 |
| **Cluster 3: CYP, SVN, LTU, ISR, POL, ARM, MDA** | | | | | | |
| median_stringency_index | 1.98 | 80.60 | 73.97 | 6.31 | 10.27 | 0.047 |
| gdp_per_capita | -2.62 | 23952.36 | 35933.75 | 10922.24 | 14062.66 | 0.009 |
| **Cluster 4: NOR, ISL, FIN, CZE, EST, LVA, AUT, SWE** | | | | | | |
| median_stringency_index | -3.71 | 62.73 | 73.97 | 6.03 | 10.26 | 2e-04 |
| **Cluster 5: NLD, DNK, IRL** | | | | | | |
| Mean_density_pig | 3.19 | 10634.85 | 2858.42 | 6732.28 | 4415.29 | 0.001 |
| gdp_per_capita | 2.35 | 54163.45 | 35933.75 | 9342.52 | 14062.67 | 0.019 |
| **Cluster 6: BEL, ESP, FRA, ITA** | | | | | | |
| covid19_cumul_death | 4.03 | 606.44 | 180.50 | 136.48 | 226.53 | 5.51e-05 |
| Mean_density_wb | 3.54 | 1.33 | 0.57 | 0.23 | 0.46 | 4e-04 |
| median_stringency_index | 2.05 | 83.79 | 73.97 | 3.02 | 10.26 | 0.04 |

ARM: Armenia; AUT: Austria, BEL: Belgium; CYP: Cyprus; CZE: Czech Republic; DNK: Denmark; ESP: Spain; EST: Estonia; FIN: Finland; FRA: France; ISL: Iceland; ISR: Israel; ITA: Italy HUN: Hungary; IRL: Ireland; LVA: Latvia; LTU: Lithuania; MDA: Moldova; NLD: Netherlands; NOR: Norway; POL: Poland; ROU: Romania; SVN: Slovenia; SWE: Sweden.
