# Supplementary material for: Surveillance and Control of African Swine Fever in the Early Phase of the COVID-19 Pandemic, March-May 2020: A Multi-Country E-Survey
Source: Front Vet Sci. 2022 Jun 6;9:867631. doi: 10.3389/fvets.2022.867631 (PMC9238323; doi:10.3389/fvets.2022.867631)
Supplement: Supplementary Material 1 — PDF version of the Google Forms of the questionnaire sent for this study. [file Data_Sheet_1.zip › Supplementary Material 9.DOCX]

Supplementary Material 9

**Respondents´ clusters obtained using the hierarchical clustering on principal components (HCPC) algorithm on the first four dimensions of the multiple correspondence analysis (MCA).** Clusters group respondents using their responses to the Likert scale items describing their perception of the impact of the COVID-19 PHSMs on the surveillance and control of ASF in their countries.

The column “Cla/Mod” indicates what percentage of all individuals with this modality are in this cluster. The column “Mod/Cla” indicates what percentage of all individuals in the cluster have this modality. The column “Global” indicates the percentage of subjects out of the total population with this modality. The column “v.test” represents the statistical value used to determine the significance of the variables describing the group (a positive value indicates an over-representation of the modality under consideration; a negative value represents an under-representation).

| **Response to Likert scale items** | **Cla/Mod** | **Mod/Cla** | **Global** | **p.value** | **v.test** |
| --- | --- | --- | --- | --- | --- |
| **Cluster 1: AUT1, HUN, POL, FIN, CYP, EST, ISL, SVN, ARM, ITA, BEL3, FRA** | | | | | |
| op.no.impact.surv = *op.no.impact.surv_Agree* | 93.7 | 100 | 59.26 | 9e-07 | 4.91 |
| op.better.prepare = *op.better.prepare_Agree* | 85.7 | 80 | 51.85 | 0.0016 | 3.15 |
| op.border.restr.risk.decr = *op.border.restr.risk.decr_Agree* | 75.0 | 80 | 59.26 | 0.021 | 2.31 |
| op.no.impact.surv = *op.no.impact.surv_Disagree* | 0.0 | 0 | 18.52 | 0.01 | -2.58 |
| op.better.prepare = *op.better.prepare_Disagree* | 0.0 | 0 | 22.22 | 0.003 | -2.95 |
| op.no.impact.surv = *op.no.impact.surv_Neutral* | 0.0 | 0 | 22.22 | 0.003 | -2.95 |
| **Cluster 2: ISR, ROU, BEL2, NLD, LTU, ESP, LVA, SWE** | | | | | |
| op.no.impact.surv = *op.no.impact.surv_Disagree* | 100.0 | 62.5 | 18.52 | 7e-04 | 3.39 |
| op.better.prepare = *op.better.prepare_Disagree* | 83.3 | 62.5 | 22.22 | 0.004 | 2.89 |
| op.no.impact.surv = *op.no.impact.surv_Agree* | 6.25 | 12.5 | 59.26 | 0.0025 | -3.02 |
| **Cluster 3: AUT2, BEL1, MDA, CZE** | | | | | |
| op.no.impact.surv = *op.no.impact.surv_Neutral* | 66.6 | 100 | 22.22 | 0.0008 | 3.33 |
| op.impede.usual.action = *op.impede.usual.action_Neutral* | 57.14 | 100 | 25.96 | 0.002 | 3.09 |
| op.better.prepare = *op.better.prepare_Neutral* | 42.86 | 75 | 25.96 | 0.044 | 2.01 |
| op.dens.wild.boars.incr = *op.dens.wild.boars.incr_Neutral* | 42.86 | 75 | 25.96 | 0.044 | 2.01 |
| op.better.prepare = *op.better.prepare_Agree* | 0.0 | 0 | 51.85 | 0.041 | -2.05 |
| op.dens.wild.boars.incr = *op.dens.wild.boars.incr _Disagree* | 0.0 | 0 | 55.55 | 0.028 | -2.19 |
| op.no.impact.surv = *op.no.impact.surv_Agree* | 0.0 | 0 | 59.26 | 0.019 | -2.35 |
| op.impede.usual.action = *op.impede.usual.action_Disagree* | 0.0 | 0 | 66.66 | 0.007 | -2.69 |

ARM: Armenia; AUT: Austria, BEL: Belgium; CYP: Cyprus; CZE: Czech Republic; DNK: Denmark; ESP: Spain; EST: Estonia; FIN: Finland; FRA: France; ISL: Iceland; ISR: Israel; ITA: Italy HUN: Hungary; IRL: Ireland; LVA: Latvia; LTU: Lithuania; MDA: Moldova; NLD: Netherlands; NOR: Norway; POL: Poland; ROU: Romania; SVN: Slovenia; SWE: Sweden.
