# Supplementary figures and images for: Surveillance and Control of African Swine Fever in the Early Phase of the COVID-19 Pandemic, March-May 2020: A Multi-Country E-Survey
Source: Front Vet Sci. 2022 Jun 6;9:867631. doi: 10.3389/fvets.2022.867631 (PMC9238323; doi:10.3389/fvets.2022.867631)

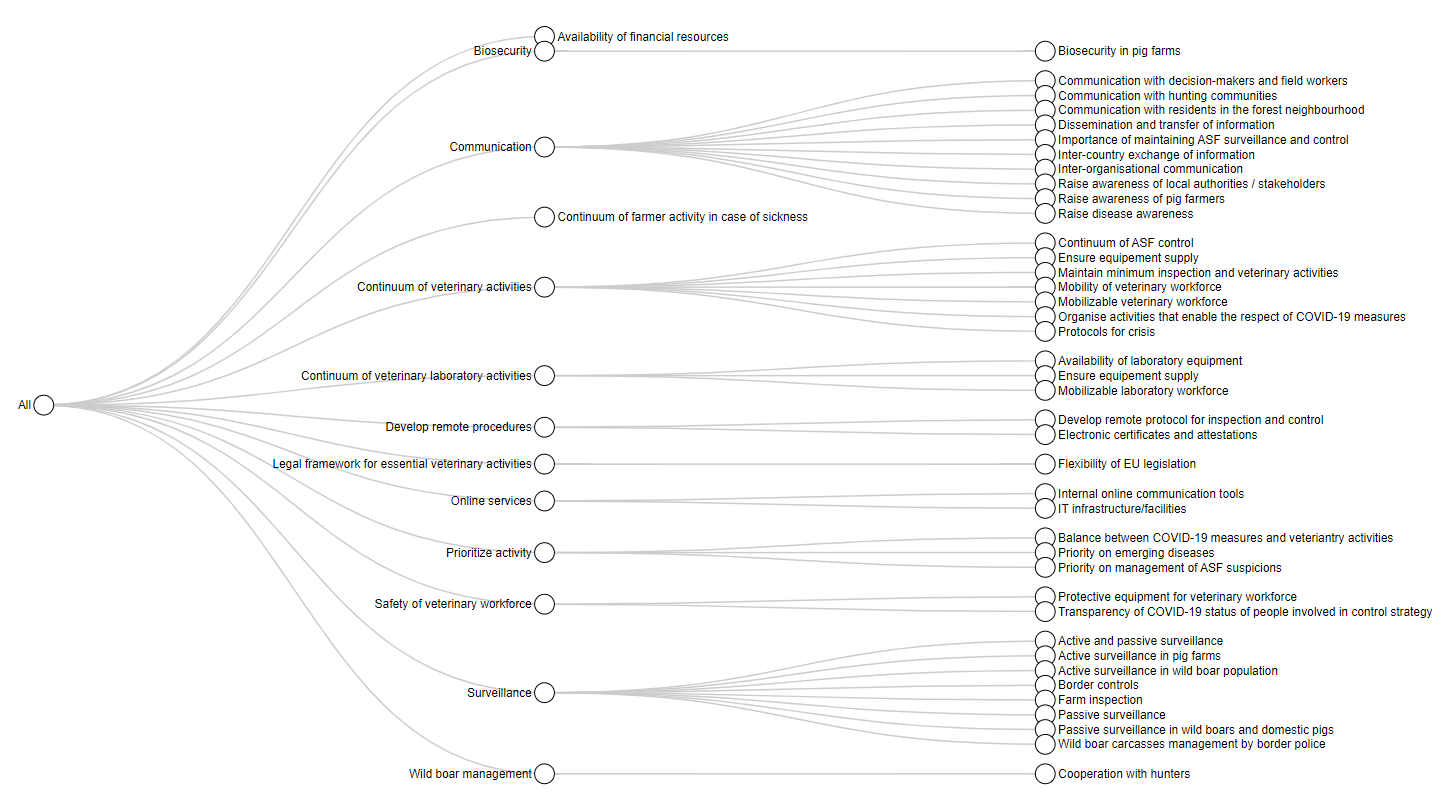

Supplement: Supplementary Material 1 — PDF version of the Google Forms of the questionnaire sent for this study. [file Data_Sheet_1.zip › Supplementary Material 10.TIF]

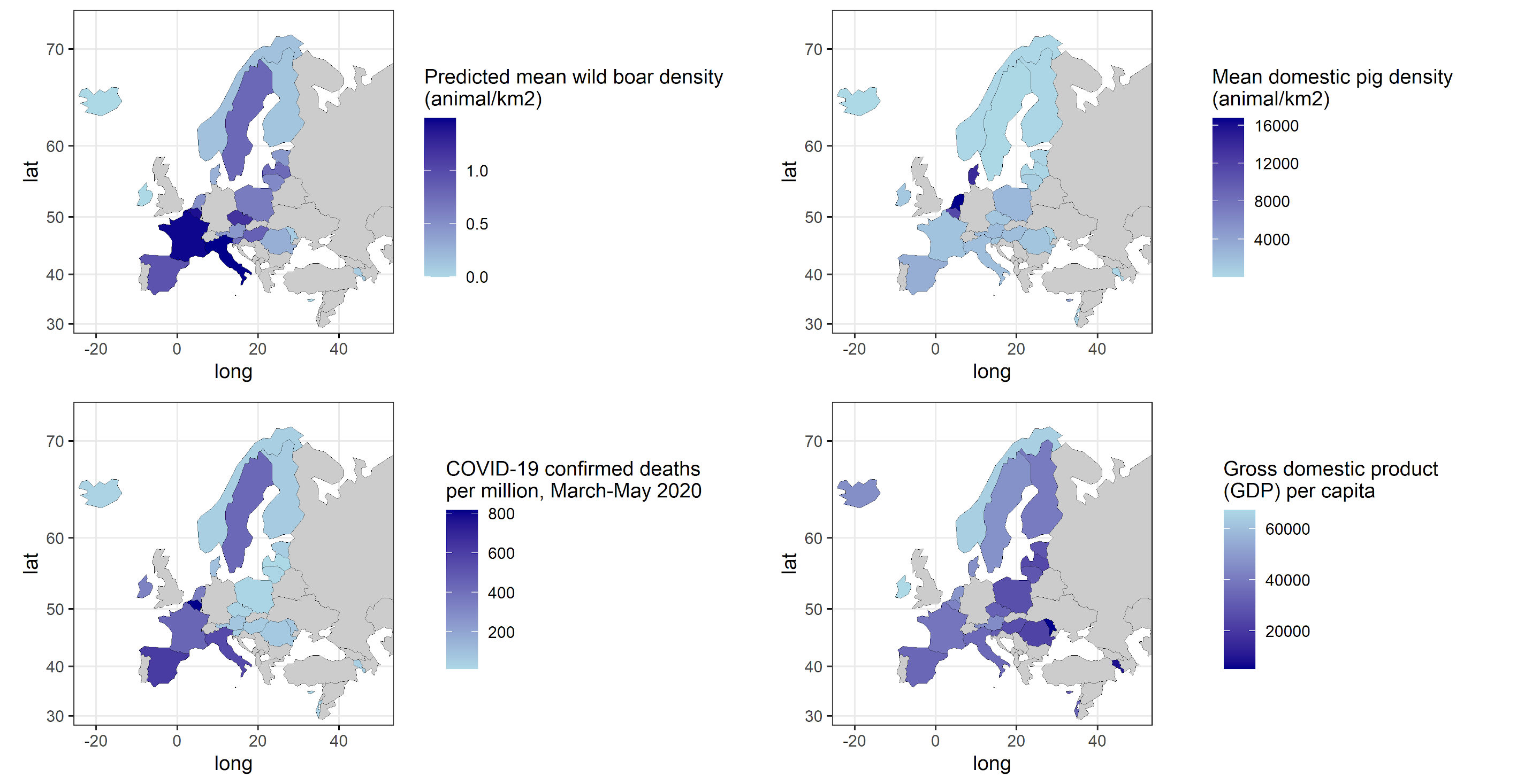

Supplement: Supplementary Material 1 — PDF version of the Google Forms of the questionnaire sent for this study. [file Data_Sheet_1.zip › Supplementary Material 2.TIFF]

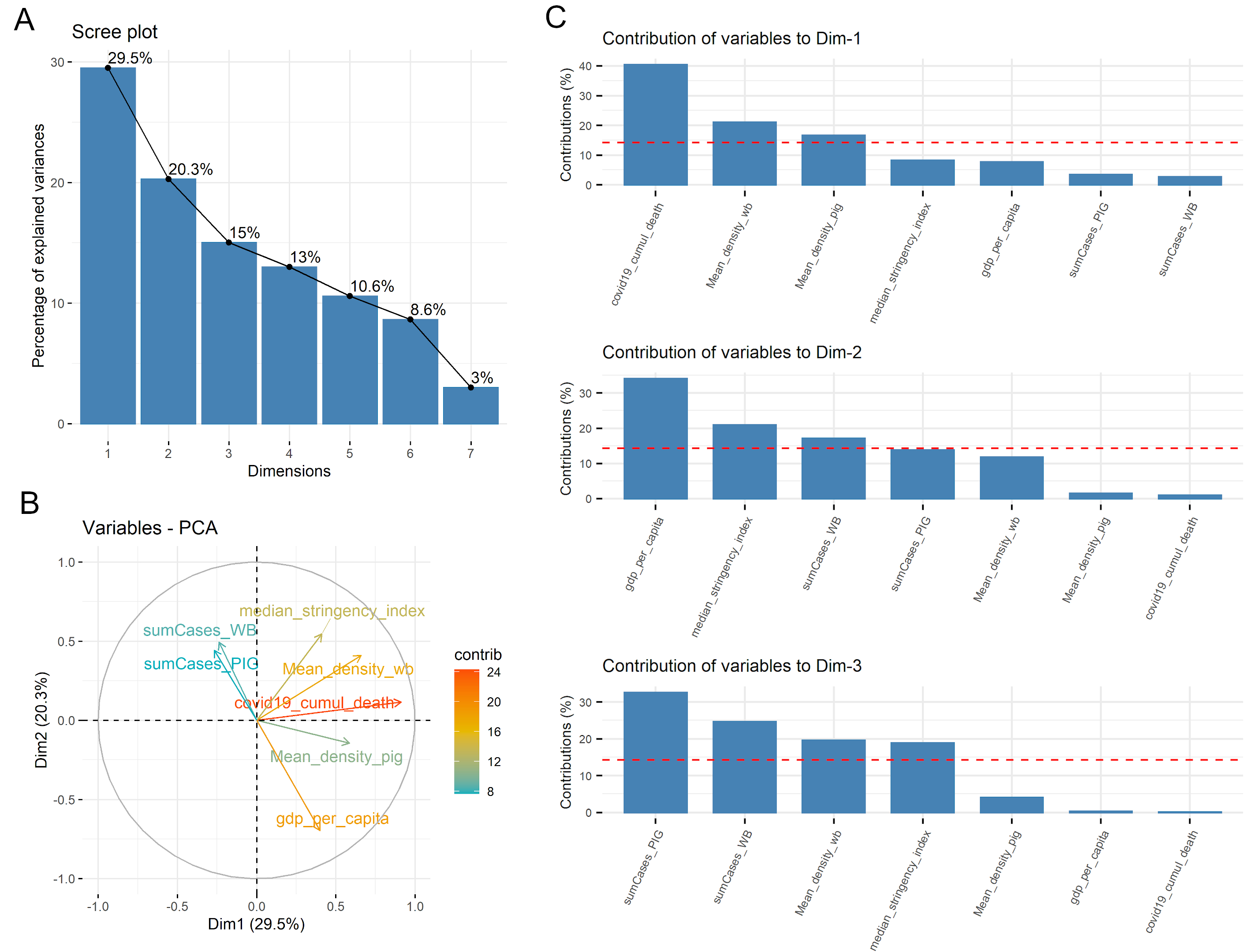

Supplement: Supplementary Material 1 — PDF version of the Google Forms of the questionnaire sent for this study. [file Data_Sheet_1.zip › Supplementary Material 3.JPEG]

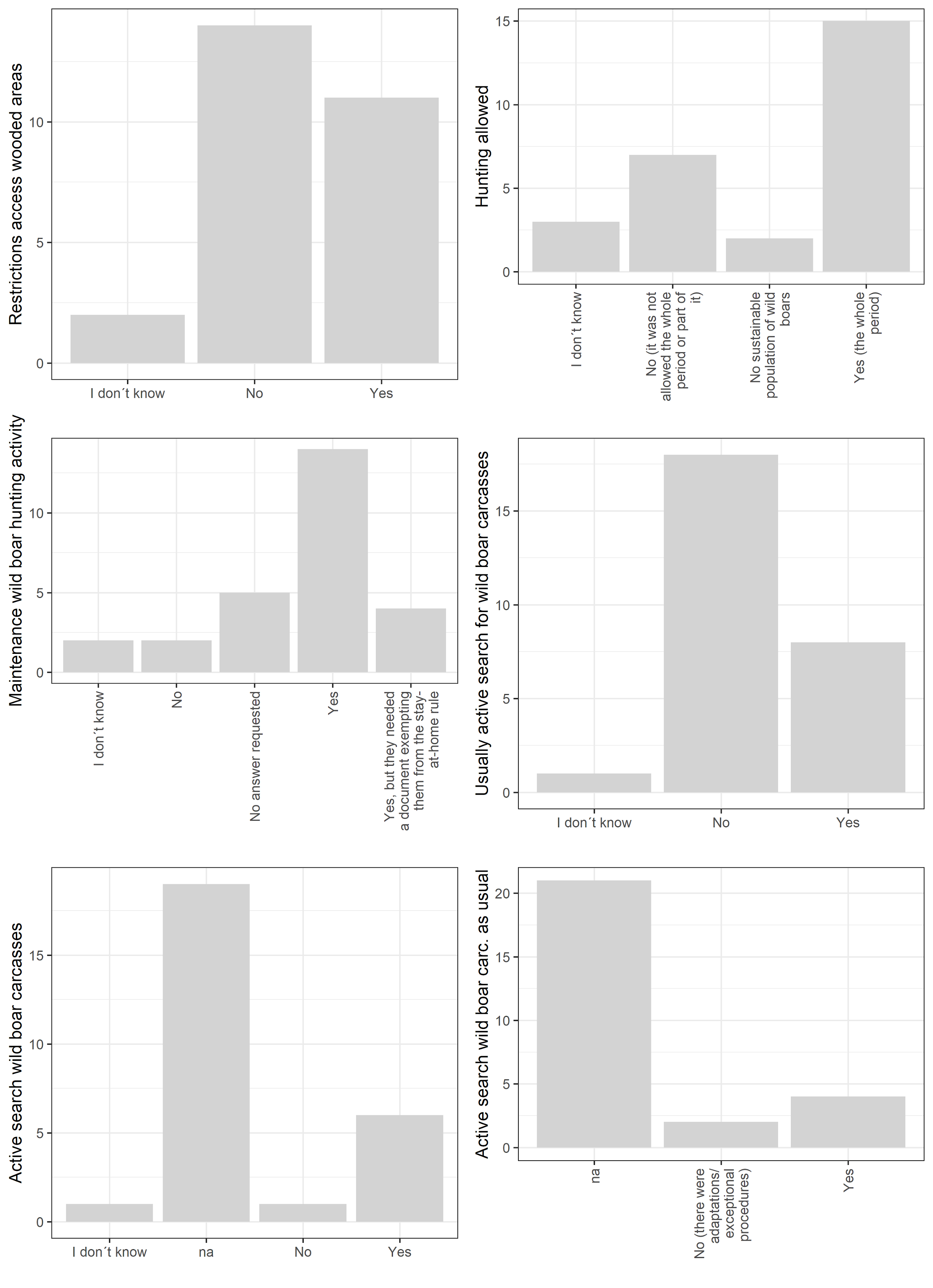

Supplement: Supplementary Material 1 — PDF version of the Google Forms of the questionnaire sent for this study. [file Data_Sheet_1.zip › Supplementary Material 5.TIFF]

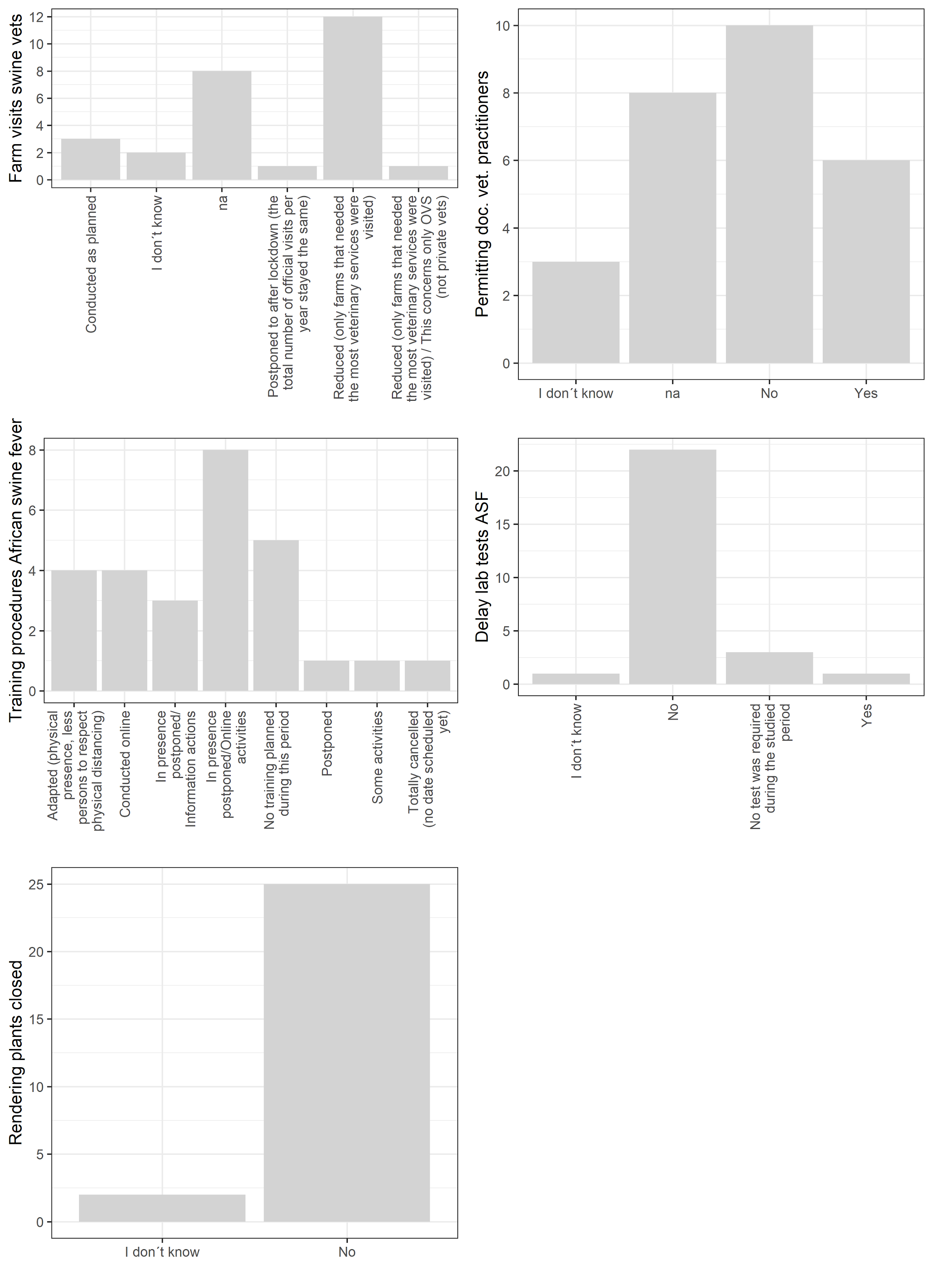

Supplement: Supplementary Material 1 — PDF version of the Google Forms of the questionnaire sent for this study. [file Data_Sheet_1.zip › Supplementary Material 6.TIFF]

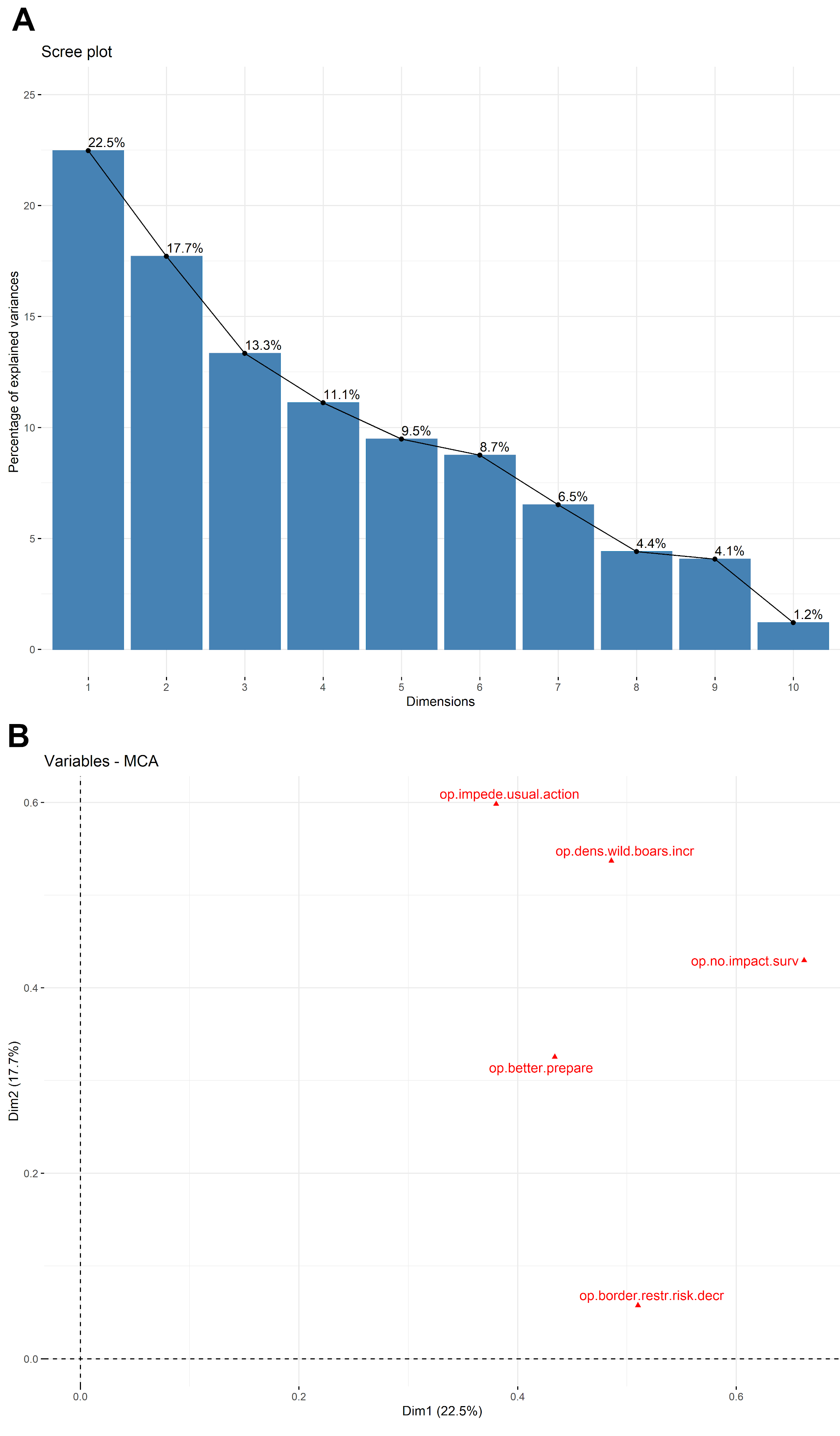

Supplement: Supplementary Material 1 — PDF version of the Google Forms of the questionnaire sent for this study. [file Data_Sheet_1.zip › Supplementary Material 7.JPEG]

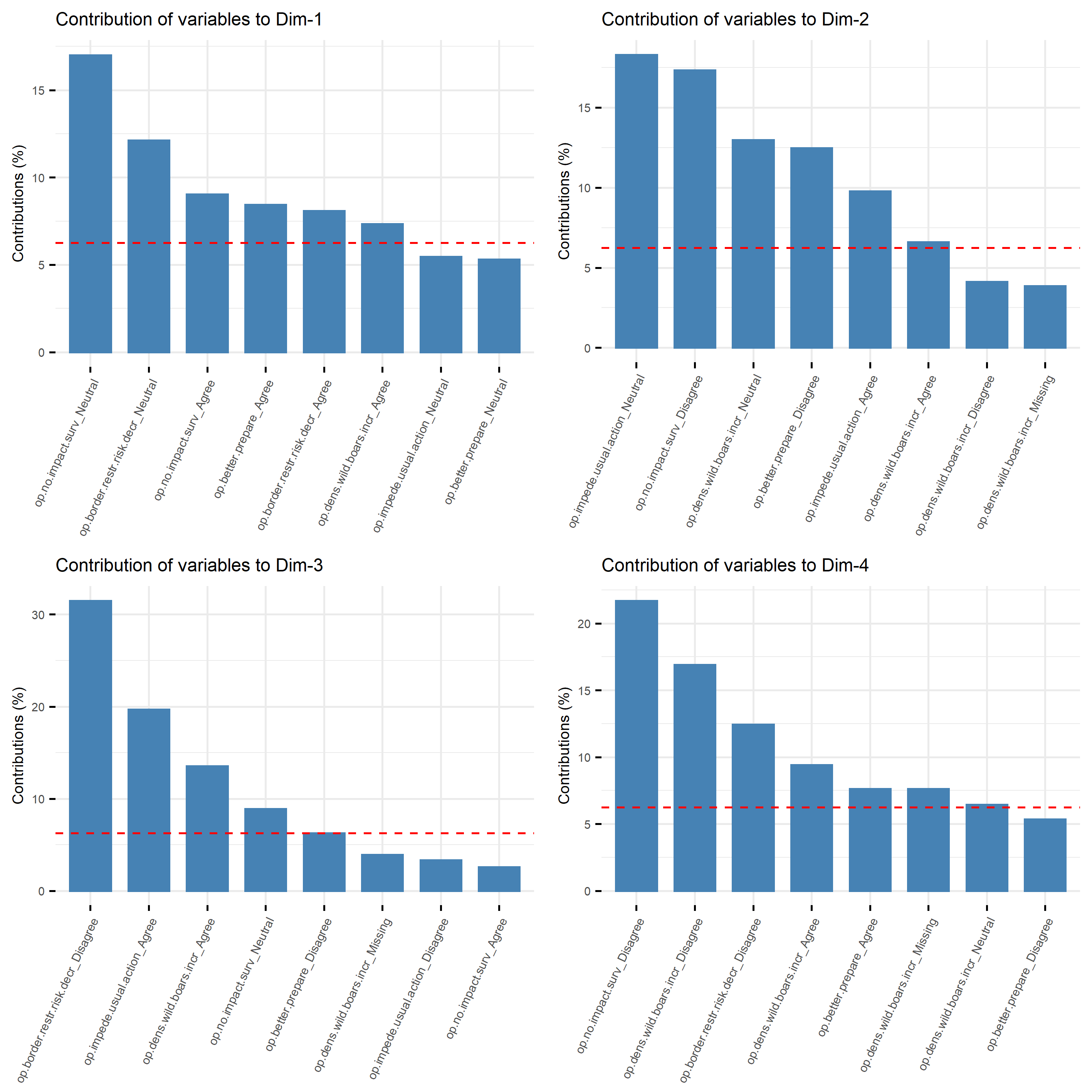

Supplement: Supplementary Material 1 — PDF version of the Google Forms of the questionnaire sent for this study. [file Data_Sheet_1.zip › Supplementary Material 8.TIFF]
